# Modeling of Effectiveness of *N*^3^-Substituted Amidrazone Derivatives as Potential Agents against Gram-Positive Bacteria

**DOI:** 10.3390/molecules29102369

**Published:** 2024-05-17

**Authors:** Małgorzata Ćwiklińska-Jurkowska, Renata Paprocka, Godwin Munroe Mwaura, Jolanta Kutkowska

**Affiliations:** 1 Department of Biostatistics and Theory of Biomedical Systems, Faculty of Pharmacy, Collegium Medicum in Bydgoszcz, Nicolaus Copernicus University in Toruń, Jagiellońska Str. 15, 85-067 Bydgoszcz, Poland; mjurkowska@cm.umk.pl; 2 Department of Organic Chemistry, Faculty of Pharmacy, Collegium Medicum in Bydgoszcz, Nicolaus Copernicus University in Toruń, Jurasza Str. 2, 85-089 Bydgoszcz, Poland; 3 Department of Pharmaceutical Chemistry, Pharmaceutics and Pharmacognosy, Faculty of Health Sciences, University of Nairobi, KNH, Nairobi P.O. Box 2149-00202, Kenya; 4 Department of Genetics and Microbiology, Institute of Biological Sciences, Maria Curie-Skłodowska University, Akademicka Str. 19, 20-033 Lublin, Poland

**Keywords:** antibacterial activity, amidrazones, Gram-positive bacteria, GLMs, LASSO, least-angle regression, stepwise, variables selection, triazoles, cyclic imides

## Abstract

Prediction of the antibacterial activity of new chemical compounds is an important task, due to the growing problem of bacterial drug resistance. Generalized linear models (GLMs) were created using 85 amidrazone derivatives based on the results of antimicrobial activity tests, determined as the minimum inhibitory concentration (MIC) against Gram-positive bacteria: *Staphylococcus aureus*, *Enterococcus faecalis*, *Micrococcus luteus*, *Nocardia corallina*, and *Mycobacterium smegmatis*. For the analysis of compounds characterized by experimentally measured MIC values, we included physicochemical properties (e.g., molecular weight, number of hydrogen donors and acceptors, topological polar surface area, compound percentages of carbon, nitrogen, and oxygen, melting points, and lipophilicity) as potential predictors. The presence of R1 and R2 substituents, as well as interactions between melting temperature and R1 or R2 substituents, were also considered. The set of potential predictors also included possible biological effects (e.g., antibacterial, antituberculotic) of tested compounds calculated with the PASS (Prediction of Activity Spectra for Substances) program. Using GLMs with least absolute shrinkage and selection (LASSO), least-angle regression, and stepwise selection, statistically significant models with the optimal value of the adjusted determination coefficient and of seven fit criteria were chosen, e.g., Akaike’s information criterion. The most often selected variables were as follows: molecular weight, PASS_antieczematic, PASS_anti-inflam, squared melting temperature, PASS_antitumor, and experimental lipophilicity. Additionally, relevant to the bacterial strain, the interactions between melting temperature and R1 or R2 substituents were selected, indicating that the relationship between MIC and melting temperature depends on the type of R1 or R2 substituent.

## 1. Introduction

The increasing number of bacterial infections is one of the challenges of modern medicine [1]. Searching for new therapeutic substances remains an actual question and task for many scientists. Despite huge developments in medicine, chemistry, and biochemistry, there are still rare diseases without effective therapies so far [2]. At the same time, the increasing drug resistance of pathogenic bacterial strains creates the risk that, in a few years, we will be left without an effective weapon against these microorganisms [3,4]. Different bacterial strains exhibit varying resistance to widely used drugs [5,6].

Bacterial drug resistance mechanisms have evolved due to the presence of selective pressure. The main known mechanisms of resistance include the limitation of drug absorption, modification of drug targets, drug inactivation, and active drug efflux. Antibiotic resistance in Gram-positive cocci is still a current problem, and the selection pressure of antibiotics is one of the most important factors contributing to its spread. Methicillin-resistant *Staphylococcus aureus* (MRSA) and vancomycin-resistant enterococci (VRE), which cause nosocomial infections, are of particular concern. Additionally, many Gram-positive bacteria often have high natural intrinsic antimicrobial resistance. The genetic and biochemical basis of antimicrobial resistance in these groups of bacteria is diverse and often varies within genera and/or species [7]. Therefore, effectively treating resistant bacterial infections is an important problem in contemporary medicine. Many antibiotics are no longer sufficiently effective, thus prompting the examination of the antibacterial activity of compounds isolated from plants [8,9] or forest-derived soil microorganisms [10] against significant bacteria.

Amidrazone derivatives are known for their wide biological activity, including antibacterial, antifungal, anti-inflammatory, cytoprotective, and anticancer effects [11]. Previous studies have demonstrated that unsubstituted amidrazones and their chloride or bromide salts exhibit good antibacterial activity. Due to our experience in the synthesis of *N*^3^-substituted amidrazone derivatives, compounds such as acyclic derivatives [12], 1,2,4-triazole derivatives [13] and cyclic imides [14] were selected for this research. In this study, we attempted to determine the influence of the R1 and R2 substituents and other structural factors of new *N*^3^-amidrazone derivatives on their antibacterial against selected strains of Gram-positive bacteria.

The search for new drugs is a long-standing and expensive process, with as many as 90% of promising substances being discarded for failing to meet the strict demands of several clinical trial phases [15]. Multidimensional statistical methods or machine learning procedures might be useful tools in the earliest stages of drug design [5,16,17]. These modern methods are increasingly used in areas such as the financial sector, energy sector, entertainment, and health care, in addition to the academic environment [18]. Artificial intelligence methods are increasingly used in structure-based drug discovery [19].

Innovative machine learning methods can be helpful in medicinal chemistry. These types of tools are increasingly used at the earliest stages of drug design, and their effectiveness is a key benefit of this approach. This method was used to study the activity of synthetic and natural small molecules and peptides against Gram-negative and Gram-positive bacteria and mycobacteria, including multidrug-resistant strains [5]. Currently used antibiotics act on one of the pathways necessary for the survival of bacterial cells, including cell wall synthesis and the biosynthesis of nucleic acids or proteins. Due to the rapidly developing antibiotic resistance (e.g., production of enzymes inactivating antibiotics, modifications in the targeted pathways), it is important to select new biochemical and therapeutic targets for antibacterial drugs [5,20]. An interesting approach is to block the biosynthesis of peptidoglycan, the main component of the bacterial cell wall, not by targeting membrane-bound extracellular enzymes but at the cytoplasmic stage of biosynthesis by inhibiting Mur enzymes, which are essential for bacterial survival [21].

A deep neural network was applied to find molecules that are structurally divergent from conventional antibiotics and display bactericidal activity against a wide phylogenetic spectrum of bacterial strains [22]. Various types of data serve as the basis of analysis using machine learning methods, including omics data [15]. Generalized linear models (GLMs) were applied to predict minimum inhibitory concentrations (MICs) based on growth curves [23].

On the contrary, we decided to model the values of MIC for five bacterial stains based on the results of our experiments, supplemented with theoretical data describing chemical structure, using a GLM [24,25]. While GLMs with main effects and interaction analysis have been applied by other authors, they focused on plant drugs against *Pseudomonas fluorescens* [8] and examined differences between groups and incubation times.

In the synthesis of new chemical compounds, it is essential to theoretically assess their biological properties, such as antibacterial activity, anti-inflammatory properties, anti-cancer potential, and others. Developing statistical models using measurements from experiments and theoretical values associated with chemical structure characterization may prove useful for this purpose. We are interested in identifying crucial factors for models that could facilitate the design of new chemical compounds with potential antibacterial activity.

The presented work aimed to create a model for the antibacterial activity of amidrazone derivatives by evaluating their MICs using generalized linear models (GLMs). Models of growth inhibition by eighty-five *N*^3^-substituted amidrazone derivatives were examined for the following five strains of Gram-positive bacteria: *Staphylococcus aureus*, *Enterococcus faecalis*, *Micrococcus luteus*, *Nocardia corallina*, and *Mycobacterium smegmatis*.

## 2. Results

**Figure 1:**
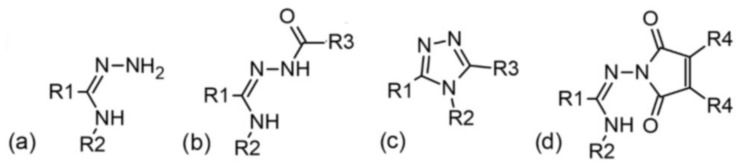
General formulas of (**a**) parent *N*^3^-substituted amidrazones, (**b**) acyclic derivatives, (**c**) 1,2,4-triazole derivatives, and (**d**) cyclic imides used to build models; where R1 = 2-pyridyl, 4-pyridyl, or phenyl; R2 = 2-pyridyl, 4-methylphenyl, 4-nitrophenyl, or phenyl; R3 and R4 depend on the anhydride used in the synthesis.

The models for predicting MICs, an in vitro measure of the pharmacodynamic potency of the drug [26], were developed for five bacterial strains. Experimental and theoretical data from 85 *N*^3^-substituted amidrazone derivatives, including derivatives of 1,2,4-triazole and cyclic imides (general structures are shown in Figure 1, full formulas in Figures S1–S3 in the Supplementary Materials), were used to build models.

The influence of R1 and R2 substituents was analyzed in the models, but due to the large number of categories causing greater computational complexity, the influence of R3 and R4 substituents was omitted.

Values of MIC for the five bacterial strains were the explained variables in building GLMs. For each Gram-positive bacterial strain, least absolute shrinkage and selection operator (LASSO), least-angle regression (LAR) and stepwise selections were applied to create GLMs. The meaning of potential variables as well as methods of their calculation are given in Table 1. Variables such as molecular weight (MW), theoretical measure of lipophilicity (miLOGP), donors of hydrogen (Donors_H), acceptors of hydrogen (Acceptors_H), and topological polar surface area (TPSA) [27] were calculated by Molinspiration online software [28] using the Simplified Molecular Input Line Entry System (SMILES) codes of studied compounds. SMILES codes and the percentages of carbon, nitrogen, and oxygen of compounds were generated using the ChemSketch program. Variables denoting biological activity such as antibacterial (PASS_antibact), anti-inflammatory (PASS_anti-inflam), antieczematic (PASS_antieczematic), antitumor (PASS_antitumor), antituberculosis (PASS_antituberculosi) were calculated with PASS Online software version 2.0 [29,30], using the SMILES codes. Variables such as PASS_anti*PASS_antib, meltingTemp*R1_substituent, meltingTemp*R2_substituent, meltingTemp*R2_substituent, and meltingTe*meltingTem were calculated directly by the Statistical Analysis System (SAS) [31]. The remaining two variables melting point (meltingTemp) and experimental lipophilicity (RMoExper) were collected through experiments. Experimental lipophilicity values were evaluated using reversed-phase thin-layer chromatography (TLC) [32]. Discrete descriptors such as R1 and R2 were incorporated into this study as binary dummy variables.

Estimated parameters for the best models, according to fit and performance measures such as adjusted determination coefficient (Adj R^2^), Akaike’s information criterion (AIC), corrected Akaike’s information criterion (AICC), Mallows’ C(p) statistic, two information criteria as Sawa’s Bayesian Information Criterion (BIC) and Schwarz Bayesian Criterion (SBC), predicted residual sum of squares (PRESS), and mean square error on the validation set (described in the Section 4) are presented in Table 2, Table 3, Table 4, Table 5, Table 6 and Table 7, while the remaining Tables S1–S24 can be found in the Supplementary Materials. In Tables S2–S16 fit statistics for models are given with F-values and *p*-values from analysis of variance (ANOVA) results.

The models for the analyzed bacterial strains differed in the number of selected important variables and the quality of prediction, and measured various criteria including adjusted coefficient of determination R^2^ and other fit and performance criteria. Therefore, the details of outcomes are presented in subsections. The unstandardized estimate for the *i*-th coefficient in the models is denoted by b_i_, while the standardized estimate for the *i*-th coefficient in the models is denoted by β_i_.

Thus, MIC (M) was modeled after the calculation of GLM selection in equations with unstandardized coefficients, as follows:

M = b_0_ + ∑ b_i_ x_i_ (1)

or with standardized coefficient (without intercept) as follows:

M = ∑ β_i_ x_i_ (2)

where the summing is performed from one to the number of selected predictors, *p*.

Unstandardized coefficients are useful in the biochemical interpretation of models obtained as GLM results (given in the left part of Table 2, Table 3, Table 4, Table 5, Table 6 and Table 7). When assuming a model without an interaction term, the value of the unstandardized coefficients denotes the change in the MIC variable (denoted as M in the above equations) with a one-unit increment in the explanatory variable (main effect) x_i_. More precisely, positive values of the unstandardized coefficient estimate, b_i_, of the numerical predictive variable, x_i_, result in an increase in the MIC value on average by b_i_ when the explaining variable is changed by one unit. Conversely, negative values indicate a decrease. This also holds if this variable is the melting temperature, in the case of the absence of interaction of melting temperature with any substituent R1 or R2 in the found model.

However, explaining variables are measured by different scales, so unstandardized coefficients depend on the scales. To interpret the objective meaning of variables (or effects) from a chemical point of view, standardized coefficients in the GLM (presented numerically or graphically) are used for inference. Standardized coefficients are obtained by dividing unstandardized coefficients by standard deviations of the respective explanatory variables. For the comparison of the impact of any predicting variable x_i_ on the MIC variable, the standardized coefficients are interpreted similarly, in terms of changes measured in standard deviations. Specifically, for numerical explaining variables, the positive (negative) values of estimates of the standardized coefficients β_i_ (presented in the right part of Table 2, Table 3, Table 4, Table 5, Table 6 and Table 7) are on average associated with one standard deviation (SD) increase (decrease) of the predictor by β_i_, assuming the other variables remain unchanged (and again assuming lack of interaction of this variable with any substituent R1 or R2 in the found model).

For cases of interaction between variable x_i_ and any substituent effect, R1 or R2, in the found models, this interpretation (for both models with unstandardized and standardized estimates) becomes slightly more complicated. In such cases, coefficients b_j_ (β_j_) found in the model for the interaction term of the numerical variable x_i_ with the discrete effect should be added to b_i_ (or β_i_, respectively) to estimate the change in the MIC variable when the predictor variable x_i_ is changed by one (or 1 SD, respectively).

Furthermore, the importance of the i-th effect is measured by the absolute value of the standardized coefficient: |β_i_|. Additionally, the sign of β_i_ further indicates the direction of MIC changes when the explaining variable is changed. An independent numerical variable with a larger absolute value of standardized coefficient will have a greater impact on the predicted variable MIC. Therefore, standardized coefficients, βi, are valuable for comparing the impact of the explaining numerical variable x_i_ (effect) on MIC.

**Figure 2:**
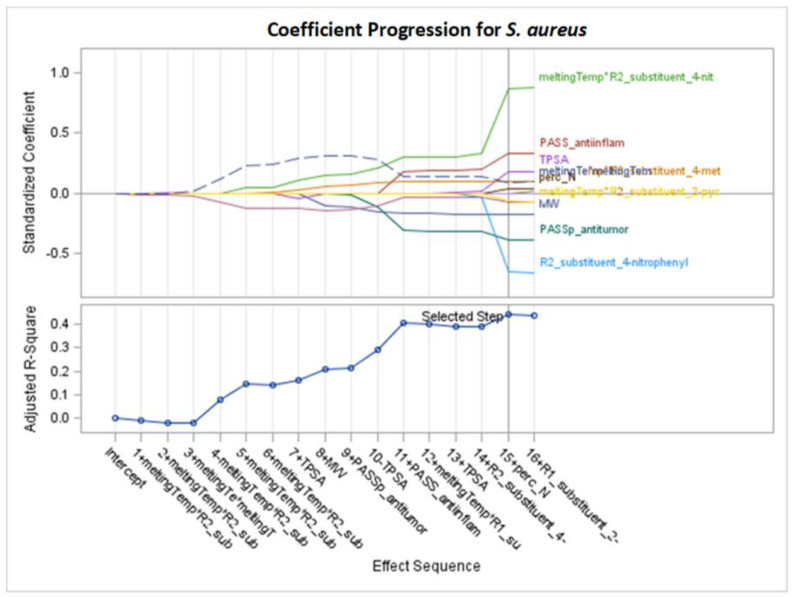
Standardized coefficients for consecutive steps of adaptive LASSO selection according to Adj R^2^, AIC, and C(p) for *S. aureus* (final: R^2^ = 0.5160, Adj R^2^ = 0.4410, *p* < 0.0001).

**Figure 3:**
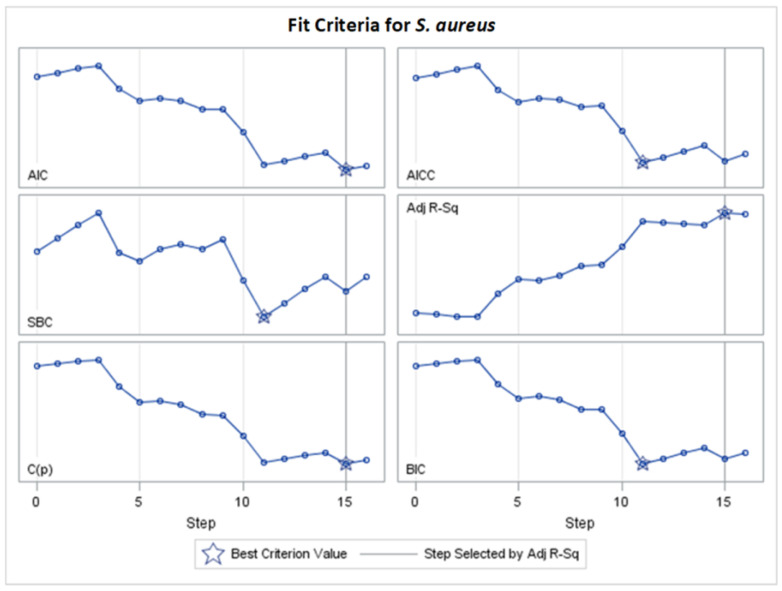
Changes in fit criteria for consecutive steps of adaptive LASSO selection according to Adj R^2^ for *S. aureus* (final: R^2^ = 0.5160, Adj R^2^ = 0.4410, *p* < 0.0001).

**Figure 4:**
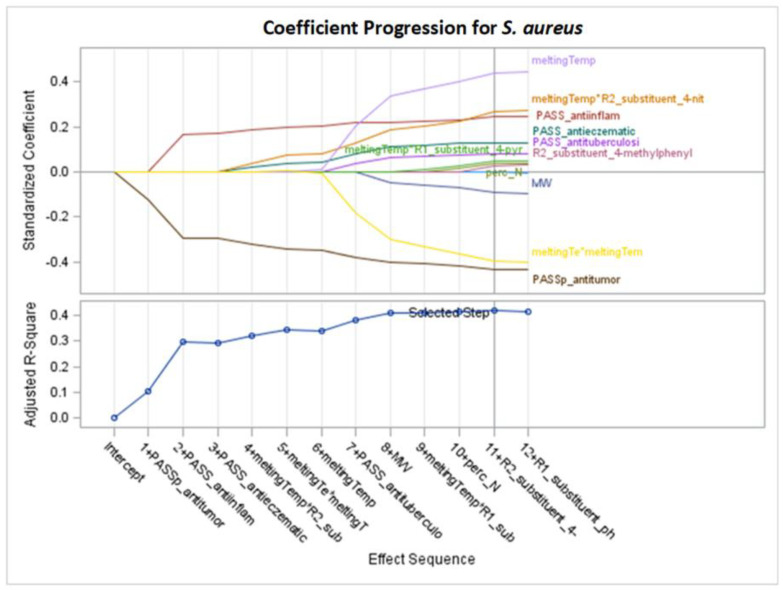
Standardized coefficients for consecutive steps of LAR selection according to Adj R^2^ for *S. aureus* (final: R^2^ = 0.4978, Adj R^2^ = 0.4200, *p* = 0.0001).

**Figure 5:**
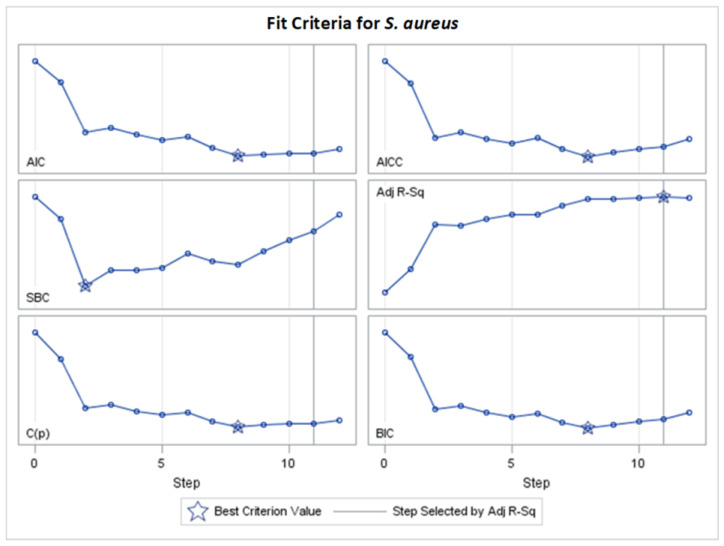
Changes in fit criteria for consecutive steps of LAR selection according to Adj R^2^ for *S. aureus* (final: R^2^ = 0.4978, Adj R^2^ = 0.4200, *p* = 0.0001).

**Figure 6:**
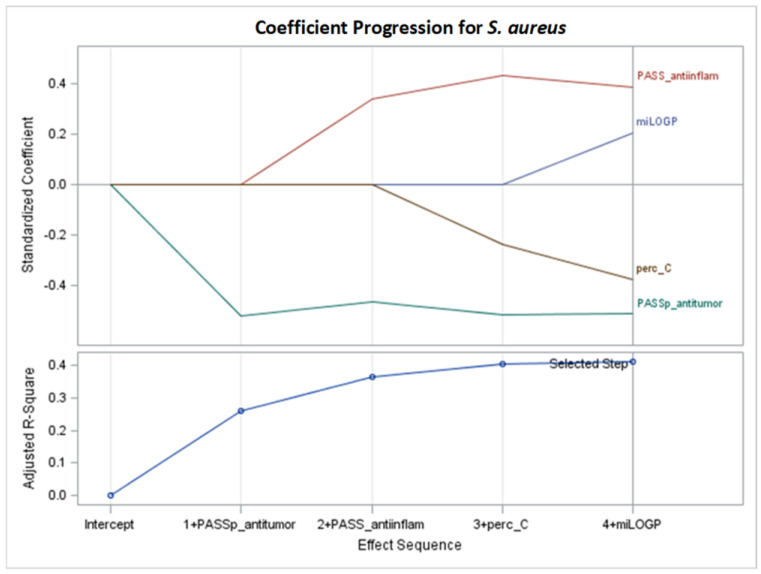
Standardized coefficients for consecutive steps of stepwise selection according to Adj R^2^, AIC, AICC, BIC, C(p), and PRESS criteria for *S. aureus* (final: R^2^ = 0.4415, Adj R^2^ = 0.4129, *p* < 0.0001).

**Figure 7:**
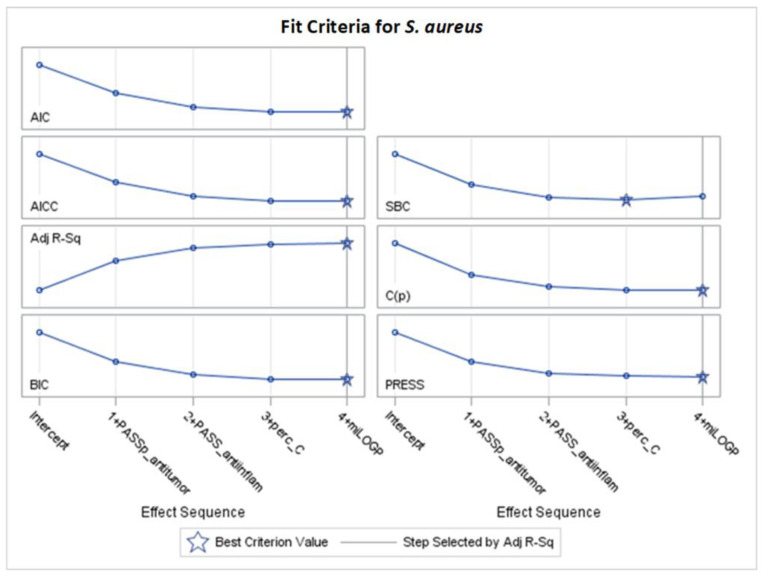
Changes in fit criteria for consecutive steps of stepwise selection according to Adj R^2^ for *S. aureus* (final: R^2^ = 0.4415, Adj R^2^ = 0.4129, *p* < 0.0001).

**Figure 8:**
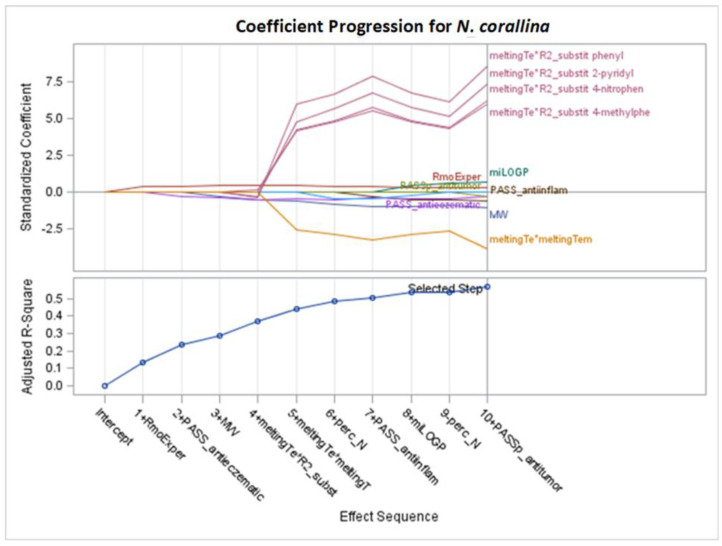
Standardized coefficients for consecutive steps of stepwise selection according to Adj R^2^, AIC, AICC, C(p), and SBC criteria for *N. corallina* (final: R^2^ = 0.6645, Adj R^2^= 0.5674, *p* < 0.0001).

**Figure 9:**
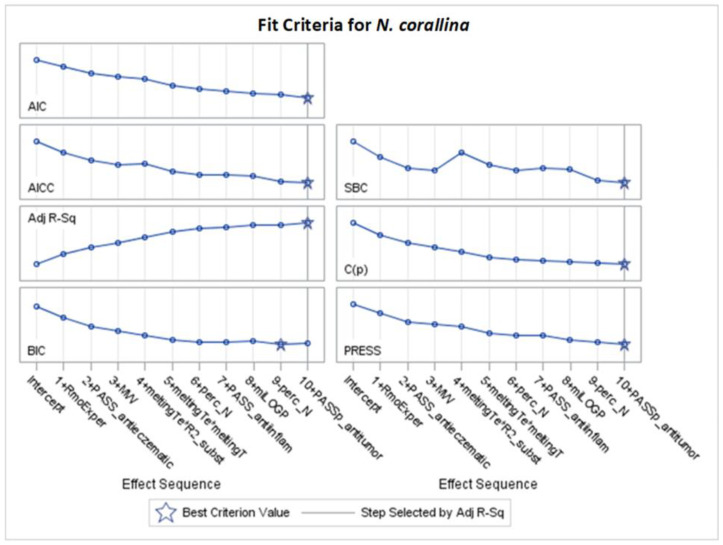
Changes in fit criteria for consecutive steps of stepwise selection according to Adj R^2^ for *N. corallina* (final: R^2^ = 0.6645, Adj R^2^= 0.5674, *p* < 0001).

**Figure 10:**
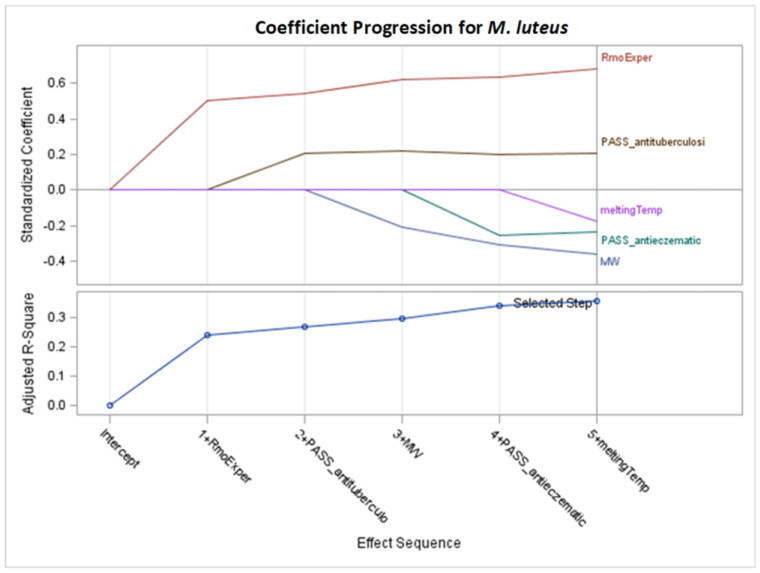
Standardized coefficients for consecutive steps of stepwise selection according to Adj R^2^, AIC, BIC, C(p), and PRESS criteria for *M. luteus* (final: R^2^ = 0.4149, Adj R^2^ = 0.3564, *p* < 0.0001).

**Figure 11:**
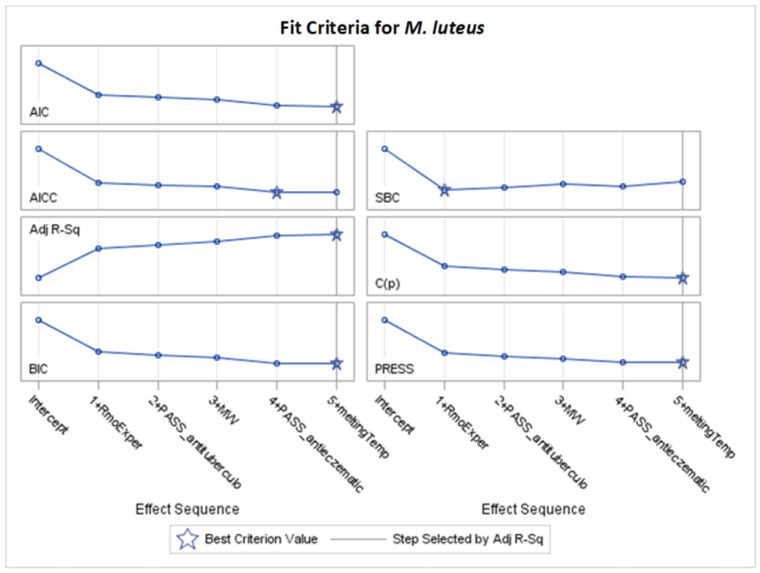
Changes in fit criteria for consecutive steps of stepwise selection according to Adj R^2^ for *M. luteus* (final: R^2^ = 0.4149, Adj R^2^ = 0.3564, *p* < 0.0001).

**Figure 12:**
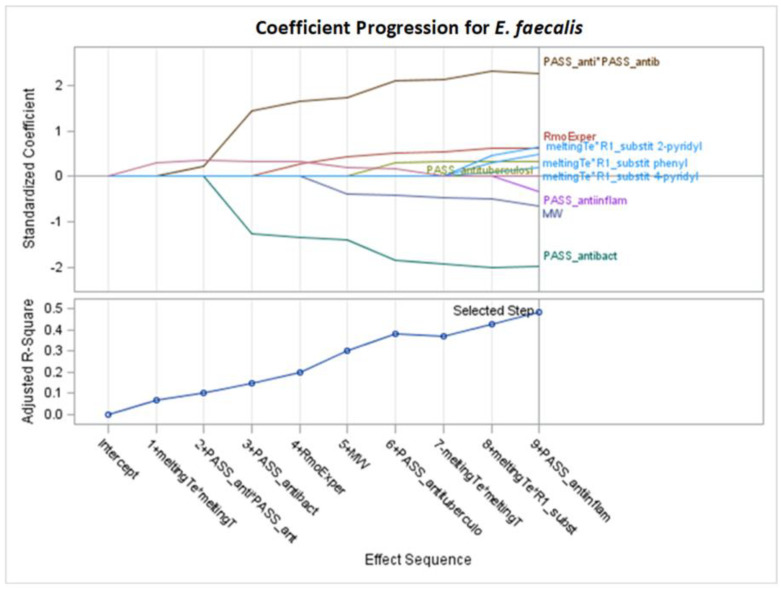
Standardized coefficients for consecutive steps of stepwise selection according to Adj R^2^, AIC, AICC, BIC, C(p), and PRESS criteria for *E. faecalis* (final: R^2^ = 0.5679, Adj R^2^ = 0.4834, *p* < 0.0001).

**Figure 13:**
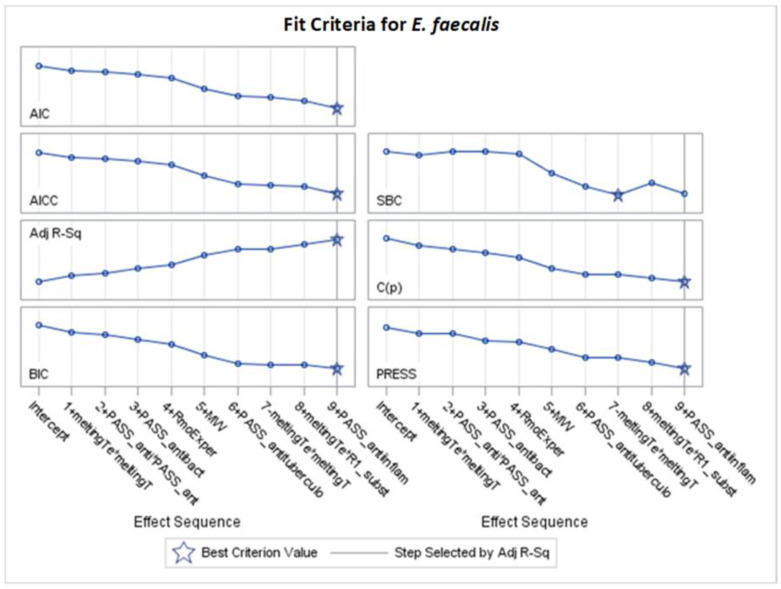
Changes in fit criteria for consecutive steps of stepwise selection according to Adj R^2^ for *E. faecalis* (final: R^2^ = 0.5679, Adj R^2^ = 0.4834, *p* < 0.0001).

Only significant models, with *p*-values of the F-statistic below the 0.05 level, were regarded in the analysis of selected effects results for the prediction of antibacterial activity, and models with the optimal value of adjusted R^2^ (Adj R^2^) and seven other fit information criteria were chosen for further consideration (Figure 2, Figure 3, Figure 4, Figure 5, Figure 6, Figure 7, Figure 8, Figure 9, Figure 10, Figure 11, Figure 12 and Figure 13, Table 2, Table 3, Table 4, Table 5, Table 6 and Table 7).

### 2.1. Models for Staphylococcus aureus

According to Tables S1–S3, considering determination coefficients R^2^ with Adj R^2^ values and other fit criteria, three selection models (LASSO, LAR, and Stepwise) for modelling the inhibition of *S. aureus* are worth considering. Therefore, these models are discussed in detail in the following three subsections: Section 2.1.1, Section 2.1.2, Section 2.1.3.

#### 2.1.1. Models for *S. aureus* Selected by Adaptive Least Absolute Shrinkage and Selection Operator LASSO

According to Table S1, the best results for *S. aureus* (n = 83) for LASSO selection were obtained using several criteria such as Adj R^2^, AIC, and C(p), creating equivalent models. Specifically, 51.6% of the variance in antimicrobial activity can be explained by the same 11 variables selected for models M1, M2, and M5 (by Adj R^2^, AIC, and C(p) criteria, respectively), while 45.4% of the variance is explained by 7 variables creating models M3 and M6 (based on the AICC and SBC criteria, respectively). Additionally, 49.8% of MIC variation is explained by 12 variables, selected according to the ASE Validation criterion, creating model M7 (Table S1).

The best models are selected for visualizing the selection steps, demonstrating how the standardized parameters change during the process. The sequential selection steps with final models are presented in Figure 2 and other analogous figures. Each line characterizes one explanatory variable and its significance in the model at each step. In the plots, we can observe at which step each variable is entered into the model or removed from the model and to what extent they have an impact on the predicted variable during the creation of model steps. Standardized coefficients show the comparable importance of variables, and the chosen step number defining the final model is denoted by a vertical line. At the end of the selection process, we can compare the magnitude and sign of standardized coefficients for variables chosen for the final model (Table 2). In Figure 2 (or in other analogous figures) the horizontal axis is described by the numbers of consecutive steps, the positive or negative sign (+ or −), indicating the addition or removal of variables, and the variable name (description of names are given in the Section 4).

The right side of Figure 2 represents the final stage of the variable selection. The effects in the 15th step can be listed from the largest standardized coefficients to the smallest. The effect names are visible from the top (where the highest positive coefficients and the most important variables are achieved) gradually down to the lowest values (negative but still important). These variables and their coefficients in the model are as follows: meltingTemp*R2_substituent_4-nitrophenyl (0.862991), PASS_anti-inflam (0.325141), TPSA (0.17846), meltingTemp*R2_substituent_4-methylphenyl (0.094897), meltingTe*meltingTem (0.090542), perc_N (0.04068), meltingTemp*R1_substituent_2-pyridyl (−0.064224), meltingTemp*R2_substituent_2-pyridyl (−0.073312), MW (−0.174756), PASS_antitumor (−0.391309), and R2_substituent_4-nitrophenyl (−0.648931). These values can also be read from Table 2 (column 5).

Figure 3 and other analogous figures serve as counterparts to Figure 2 and its analogous figures. They depict the values of all examined fit measures (Adj R^2^, AIC, AICC, BIC, C(p), SBC, ASE Val, and PRESS) corresponding to the consecutive steps of the model building presented in Figure 2 (and its analogues) with the selected criterion statistic (or criteria if few models are the same during consecutive steps). The maximum Adj R^2^ and the minimum AIC and C(p) occur at step 15, where 11 variables are chosen in the model. The same models created by the optimization of different fit criteria can be identified from Table 2 (or an analogous table) by the same finally chosen variables and their final coefficients (standardized or unstandardized). Equivalent models are also confirmed in Tables S1–S3 (by the same final fit measures in corresponding columns).

The values of unstandardized coefficients presented in Table 2 give the possibility to predict MICs for given values of variables. The interpretation of the unstandardized coefficients is as follows: it represents the change in MIC if other numerical variables are set unchanged (e.g., at their mean values).

According to the left part of Table 2 (for unstandardized coefficients), an example of the model equation (M1 = M2 = M5) for MIC can be written as follows:

M = 873.169475 − 2.046303 ∙ MW + 617.458702 ∙ PASS_anti-inflam − 1243.082270 ∙ PASS_antitumor + 4.769786 ∙ perc_N + 2.715861 ∙ TPSA − 658.793078 ∙ R2_substituent_4-nitrophenyl − 0.339383 ∙ melting-Temp*R2_substuent_2-pyridyl + 0.401398 ∙ meltingTemp*R2_substituent_4-methylphenyl + 5.050186 ∙ meltingTemp*R2_substituent_4-nitrophenyl − 0.234074 ∙ meltingTemp*R1_substituent_2-pyridyl + 0.001853 ∙ meltingTe*meltingTem.

Similarly, the equation for M with standardized coefficients (without intercept) from Table 2 may be written.

Interaction terms between meltingTemp and R1 or R2 are included in the model (interaction is marked by *). The existing interaction with R1 (or R2) means that the impact of melting temperature on MIC depends on the category of R1 (or R2) substituent. Simultaneously, the same interaction coefficient means that the impact of the R1 (or R2) substituent on MIC depends on the value of the melting temperature.

Assuming other variables remain unchanged and considering R1_substituent as 2-pyridyl, taking into account the interaction of melting temperature with R1 and R2 substituent, an increase in melting temperature by one unit of standard deviation (SD) results in the following:

1. decreases MIC by −(−0.234074 + 0.001853−0.339383) = 0.571604 for R2_substituent_2-pyridyl;
2. increases MIC by −0.234074 + 0.001853 + 0.401398 = 0.169177 for R2_substituent_4-methylphenyl;
3. increases MIC by −0.234074 + 0.001853 + 5.050186 = 4.817965 for R2_substituent_4-nitrophenyl.
4. decreases MIC by −(−0.234074−0.339383) = 0.573457 for R2_substituent_phenyl.

For other R1 substituents (4-pyridyl or phenyl), an increase in melting temperature by one unit of standard deviation (SD) results in the following:

decreases MIC by −(0.001853−0.339383) = −0.33753 for R2_substituent_2-pyridyl;

increases MIC by 0.001853 + 0.401398 = 0.403251 for R2_substituent_4-methylphenyl;

increases MIC by 0.001853 + 5.050186 = 5.052039 for R2_substituent_4-nitrophenyl;

decreases MIC by 0.001853 for R2_substituent_phenyl.

According to Figure 2 and the right part of Table 2 (standardized coefficients), the variables with the greatest impact on MIC are as follows: meltingTemp*R2_substituent_4-nitrophenyl, PASS_anti-inflam (with positive signs β = 0.862991 and 0.325141), R2_substituent_4-nitrophenyl, and PASS_antitumor (with negative signs β = −0.648931 and −0.391309). A lower MIC value indicates better experimental antibacterial activity. Therefore, for instance, an increase in PASS_antitumor has a considerable impact on improving the growth inhibition of *S. aureus*, assuming that the other ten variables remain unchanged.

The “story” of creating the final model is visible in Figure 2. For example, the report in Figure 2 indicates that TPSA is included in step 7 (with a small negative standardized coefficient) and removed in step 10, only to be added again in step 13. Consequently, in the final model, TPSA is present with a considerably positive coefficient (0.178460—see Table 2). Notably the very important R2_substituent_4-nitrophenyl (β = −0.648931) is added only in the almost final step (14). The interaction effect of meltingTemp*R2_substituent_4-nitrophenyl remains in the adaptive LASSO model until step 4 (β = 0.862991). This high coefficient indicates considerable interaction between melting temperature and the R2 substituent.

The highest mean selection percentages (87% and 82.1%) by seven criteria from 1000 bootstrap samples from the dataset involve PASS_antitumor and the interaction of melting point with R2_substituent (4-nitrophenyl) (Table 2).

#### 2.1.2. Models for *S. aureus* Selected Using the Least-Angles Regression Method

According to the determination coefficients presented in Table S2, the LAR model selection was also chosen for further analysis with different fit criteria. The LAR model of variable selection explains 49.78% of MIC variation using 11 variables according to the Adj R^2^ criterion, forming the M1 mode. A lower percentage (46.8%) of MIC variation is explained using the M2–M4 models (based on AIC, AICC, and BIC criteria, respectively) with the same eight variables selected (Table S2).

The creation of a model based on the Adj R^2^ criterion is presented in Figure 4. Optimal Adj R^2^ is obtained after 11 selection steps, where one variable is added at each step.

Figure 5 shows the standardized coefficients of all the effects selected at some step of the LAR method, plotted as a function of the step number. According to standardized coefficients (also see the right part of Table 3), the effects with the highest impact on the models for MIC are meltingTemp, meltingTemp*R2_substituent_4-nitrophenyl, PASS_anti-inflam, PASS_antieczematic (with positive coefficients β = 0.435941, 0.243939, 0.265515, and 0.127085, respectively), PASS_antitumor, and meltingTe*meltingTem (with negative coefficients β = −0.431145 and −0.394171, respectively).

In the LAR model, both the interaction between melting temperature and the R1 substituent (step 9) and the interaction between melting temperature and the R2 substituent (step 4) were selected. Interaction effects indicate that the R2 substituent or R1 substituent variable influences the relationship between melting temperature and the MIC variable. The highest mean selection percentages (86.01% and 65.09%) from eight criteria from 1000 bootstrap samples from the dataset are for MW and meltingTemp (Table 3).

#### 2.1.3. Models for *S. aureus* Selected Using Stepwise Procedure

According to Table S3, the best model with the stepwise procedure is achieved using the Adj R^2^ and five other fit criteria resulting in equivalent models. The stepwise selection method yields explanatory models for 44.15% of MIC variation, based on only four variables according to criteria, creating M1–M5 and M7 models (i.e., for Adj R^2^, AIC, AICC, BIC, C(p), and PRESS, respectively). Model M6, based on the SBC criterion with three variables selected, explains 42.56% of the variability, while model M8 (ASE Val criterion) explains 43.21% with only two variables selected, namely PASS_antitumor and PASS_anti-inflam (Table S3).

We focus on the equivalent models after only six steps of stepwise selection (Figure 6 and Figure 7) according to Adj R^2^, AIC, AICC, BIC, C(p), and PRESS criteria for *S. aureus* (Adj R^2^ = 0.4129). No interaction term is selected (Table 4, Figure 6 and Figure 7), so the interpretation of the model is straightforward. Thus, according to unstandardized coefficients (left side of Table 4), MIC (M) can be estimated as the following function of four variables:

M = 2429.212180 + 76.605993 ∙ miLOGP + 732.638700 ∙ PASS_anti-inflam +

−1628.150512 ∙ PASS_antitumor − 29.306596 ∙ perc_C.

The model for MIC can be interpreted as follows: assuming the three other variables remain unchanged, an increase of one unit in PASS_anti-inflam causes an increase of 732.638700 in MIC. Similarly, assuming the other variables remain unchanged, an increase of one unit in miLOGP leads to an increase of 76.605993 in MIC.

However, the coefficients are negative for PASS_antitumor and perc_C. Therefore, assuming another three of four variables remain unchanged, an increase of one unit in PASS_antitumor results in a decrease of 1633.785168 in MIC, or, assuming the other variables remain unchanged, an increase of one unit in perc_C leads to a decrease of 29.306596 in MIC.

It should be noted that miLOGP is chosen in the stepwise model, which is the theoretical counterpart of the experimental lipophilicity (RmoExper) that is not selected.

The highest mean selection percentage (29.9%), determined by eight criteria from 1000 bootstrap samples from the dataset, corresponds to PASS_anti-inflam (Table 4).

Summarizing the results from Section 2.1, the variables that exert the greatest impact on compound activity in inhibiting the *S. aureus* bacterial strain are commonly selected, namely PASS_antitumor and PASS_anti-inflam. Additionally, for models with more than four variables, the interaction of melting point with the R2 substituent (4-nitrophenyl) is important (Table 2, Table 3 and Table 4). The variables most frequently selected by different GLM selection models show similarities across the LASSO, LAR, and stepwise selection criteria (Table 2, Table 3 and Table 4, Figure 2, Figure 4 and Figure 6).

### 2.2. Models for Nocardia corallina

According to Tables S4–S6, the best models for *N. corallina*, achieving the optimally adjusted determination coefficients (Adj R^2^) and other fit criteria, as AIC, AICC, C(p), and SBC were obtained after stepwise selection, are presented in Table 5 and Figure 8 and Figure 9. The remaining models (LASSO and LAR) are provided in Tables S16 and S17.

Figure 8 presents the changes in standardized parameters during the selection process. The best model for *N. corallina* with eight variables (12 effects together with intercept) was obtained after 10 steps of stepwise selection based on the Adj R^2^ criterion (Figure 8 and Figure 9). The variable perc_N was included in the 6th step but removed in the 9th step. Interaction effects indicate that the R2 substituent or R1 substituent variable influences the relationship between melting temperature and MIC variable. The most important variables, according to standardized coefficients, are squared melting temperature and the interaction between melting temperature and the R2 substituent (Figure 8 and Table 5). Variables such as MW (with a negative coefficient) and miLOGP (with a positive coefficient) have slightly smaller importance. Other variables in the model, such as PASS_anti-inflam, PASS_antieczematic, and PASS_antitumor, have smaller values measured by standardized coefficients (see Table 5).

The highest mean selection percentages (61.8% and 42.5%) by eight criteria from 1000 bootstrap samples from the dataset have PASS_antieczematic and MW (Table 5).

### 2.3. Models for Micrococcus luteus

Based on Tables S7–S9, and the optimal values of Adj R^2^, AIC, BIC, C(p), and PRESS criteria, stepwise models were selected for further detailed examination. The specifics of parameter estimation for the chosen stepwise selection are presented in Figure 10 and Figure 11 and Table 6, while the parameters of the remaining models (obtained by LASSO and LAR selection) are provided in the Supplementary Materials (Tables S18 and S19).

The best models for *M. luteus* were obtained for stepwise selection according to Adj R^2^, AIC, BIC, C(p), and PRESS criteria. This set of models, including M1–M2, M4–M5, and M7, uses the same subset of five variables, explaining 41.49% of MIC variability (Adj R^2^ = 0.3564). No interaction term is selected, simplifying the interpretation of the MIC model. According to the equation provided, assuming other variables remain unchanged, an increase of one unit in any variable x_i_ causes a change in MIC equal to b_i_. For example, an increase of one unit in MW results in a decrease in MIC values by 3.248648, while a one-unit increase in RmoExper leads to an increase in MIC by 251.327244.

Selected effects with larger absolute values of standardized coefficients will have a greater influence on the dependent variable. Thus, according to the standardized coefficients in Table 6, RmoExper (with a positive coefficient), together with MW and PASS_antieczematic (with negative coefficients), have the most significant impact on the model. The highest mean selection percentage (88.91%) by eight criteria from 1000 bootstrap samples from the dataset have RmoExper (Table 6).

### 2.4. Models for Enterococcus faecalis

Based on Tables S13–S15 and optimal values of Adj R^2^, AIC, AICC, BIC, C(p), and PRESS, again, stepwise selection is chosen. For *E. faecalis*, the best model was achieved after nine steps of selection, according to the mentioned criteria. Seven variables explain 56.79% of MIC variability (Adj R^2^ = 48.34%). The following variables are selected: RmoExper, MW, meltingTe*R1_substit_2-pyridyl, PASS_antituberculosi, PASS_anti-inflam, PASS_antibact, and PASS_anti*PASS_antib.

According to the standardized coefficients, the most important is the variable PASS_antibact, both in first and second power (Table 7, Figure 12 and Figure 13). Also, MW and PASS_anti-inflam (with negative coefficients of −0.472224 and −0.329342) and RmoExper (with a positive coefficient of 0.535104) are important. The interaction of melting temperature and R1 substituent with all positive coefficients should also be noted.

The highest mean selection percentages (94.21% and 80.76%) in eight criteria from 1000 bootstrap samples from the dataset are from RmoExper and MW (Table 7).

### 2.5. Models for Mycobacterium smegmatis

For *M. smegmatis*, the fit criteria obtained for selected models are not satisfying (Tables S13–S15 for *M. smegmatis*). For example, in the best LASSO model, a rather large number of effects (12) explained only 26.46% of MIC variability (model M1, with a small value of Adj R^2^ = 0.1538, Table S13; parameter estimates in Table S22). The nine-effect LAR models M2–M5 explains 23.07% of *M. smegmatis* inhibition variability (Adj R^2^ = 0.0989, Tables S14 and S24). The selection of the remaining models using LASSO and LAR, based on the remaining criteria together with all models for stepwise selection, are not useful at all (Tables S13–S15).

Mycobacteria are evolutionarily classified as Gram-positive bacteria, but the architecture of their cell wall is more complex. The outer membrane contains a variety of lipids necessary for the survival and virulence of pathogenic species. The permeability barrier of the outer membrane is a major determinant of drug resistance for many antibiotics, especially in slow-growing mycobacteria.

It has been shown that, in *Mycobacterium smegmatis*, porins play an important role in the transport of small and hydrophilic β-lactam antibiotics through the outer membrane. Hydrophobic antibiotics like moxifloxacin, in contrast to norfloxacin, were more effective in inhibiting the growth of *Mycobacterium smegmatis*, probably due to better diffusion through the lipid membrane. Structural models showed that drug molecules that were too large (e.g., erythromycin, kanamycin, and vancomycin) did not pass through porin channels in this bacterial strain [34]. The distinctive biochemical characterization of *Mycobacterium smegmatis* between five examined bacterial strains might be reflected in different model results, including different numbers of selected variables and worse final fit measures for *M. smegmatis*.

## 3. Discussion

In papers using machine learning for drug discovery [5,17], previous authors only have divided classifications into groups—e.g., whether the drug works or not—while we modeled concrete MIC values with the selection of important variables and indications of the direction of the effect. Moreover, we modeled new chemical compounds synthesized in our laboratory, in contrast to chemical compounds from the big chemical database analyzed in [5]. Both chemical structure and experimental results were included in the selection procedures, additionally considering the interaction between structure and experimental melting point.

The models for the tested bacterial strains differed from each other; however, we can point to some variables that are important for models selected for the bacteria *S. aureus*, *E. faecalis*, *M. luteus* and *N. corallina*. The overlapping of selected variables for each of the five bacterial strains and the LASSO or LAR or stepwise selection procedure is presented in Table 2, Table 7 and Tables S16–S24, with estimated parameters according to models based on Adj R^2^, AIC, AICC, BIC, C(p), SBC, average squared error on validation set (ASE Val), and PRESS criteria.

The appearance of variables (main effects) for selected models in *S. aureus* (SA), *N. corallina* (NC), *M. luteus* (ML), *E. faecalis* (EF), and *M. smegmatis* (MS) is given in Supplementary Table S25 (summarizing findings from Table 2, Table 7 and Tables S16–S24). The sum of the model numbers with positive and negative signs occurring at least once in each of the 15 tables (for five bacterial strains and three selection procedures: LASSO, LAR, and stepwise) is presented for each main effect in the last column. According to this column, the most often selected variables are molecular weight, PASS_antieczematic, PASS_anti-inflam, squared melting temperature, PASS_antitumor, and RmoExper.

After removing the weakest models of *M. smegmatis* from this summary and analyzing 12 tables (Table 2, Table 3, Table 4, Table 5, Table 6, Table 7 and Tables S16–S21), PASS_anti-inflam, molecular weight, PASS_antieczematic, squared melting point, RmoExper, and PASS_antitumor are still the most oft-present main effects, which confirms the importance of these variables (Table S26).

Models for some bacteria selected the interaction terms. Interaction effects indicate that the categorical R1 substituent (R2 substituent) variable influences the relationship between melting temperature and MIC variable. For example, when selected for *S. aureus* LASSO models (M1–M2, M5), interactions between melting temperature with R1 and R2 substituents have an impact on MIC. For the LAR model (M1), only the interaction between melting temperature and R2 substituent is chosen. Similarly for *E. faecalis*, in selected stepwise models (M1–M5, M7), the interaction between melting temperature and R2 substituent is included. However, for *N. corallina* in selected stepwise models (M1–M3, M5–M6), the interaction between the R1 substituent and melting temperature influences the MIC.

**Figure 14:**
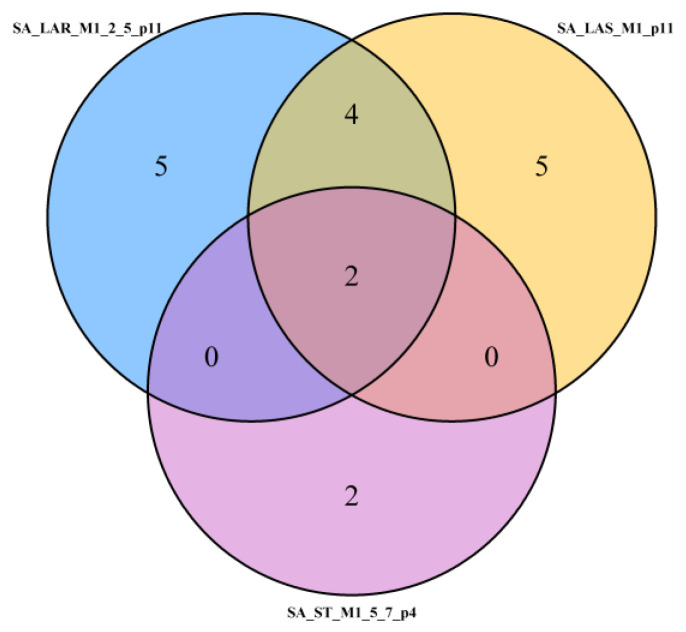
Venn diagram illustrating the common intercept PASS_anti-inflam and PASS_antitumor for three models—LASSO (LAS), LAR, and stepwise (ST) of *S. aureus* (SA). Models SA_LAR_M1_2_5_p11, SA_LAS_M1_p11, and SA_ST_M1_5_7_p4. M are models by fit criteria, *p* = #predictors selected.

**Figure 15:**
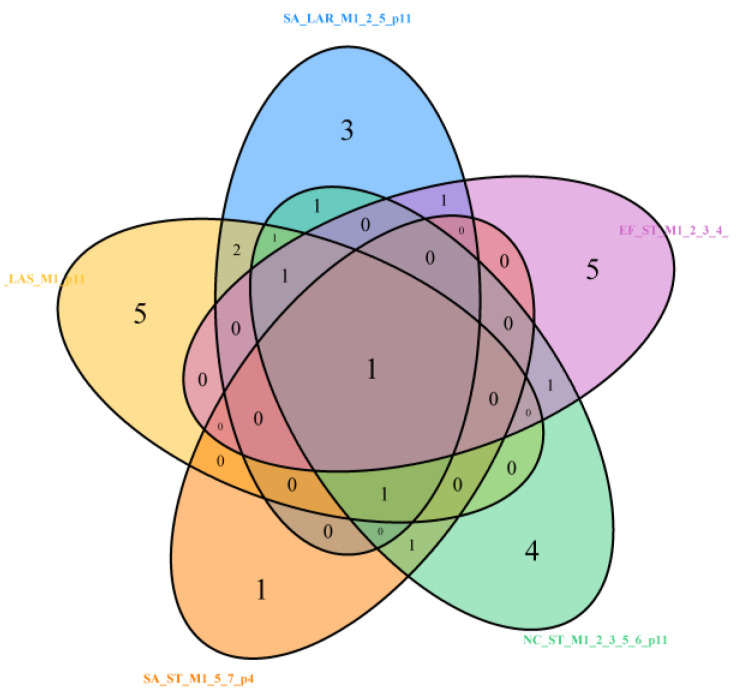
Venn diagram with common intersect PASS_anti-inflam, remaining in *S. aureus (SA)*, *N. corallina* (NC), and *E. faecalis* (EF), for SA_LAR_M1_2_5_p11, SA_LAS_M1_p11, SA_ST_M1_5_7_p4, NC_ST_M1_2_3_5_6_p11, and EF_ST_M1_2_3_4_5_7_p10 models. LASSO (LAS), LAR, and stepwise (ST). M are models by fit criteria M, p = #predictors selected.

**Figure 16:**
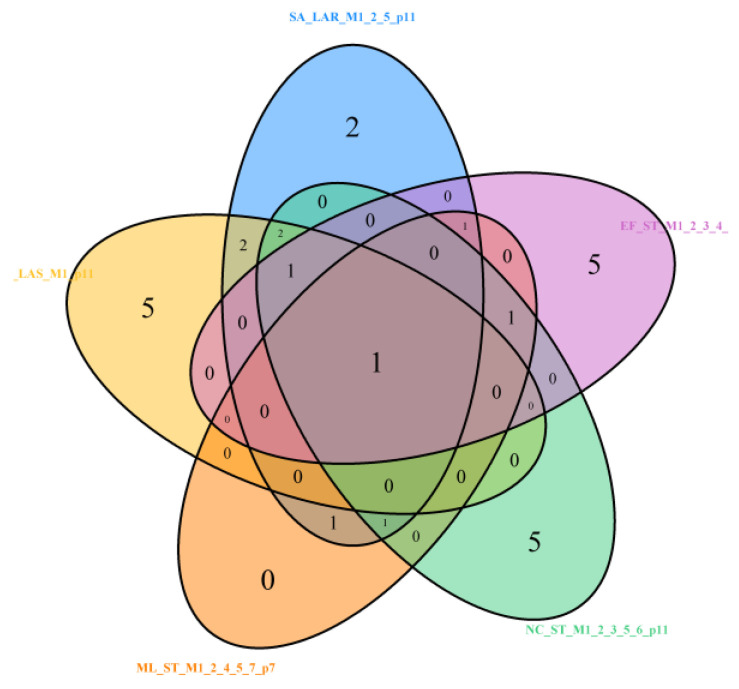
Venn diagram for models with common intersect MW in *S. aureus* (SA), *M. luteus* (ML), *N. corallina* (NC), and *E. faecalis* (EF), based on at least 7 variables. Models: SA_LAR_M1_2_5_p11, SA_LAS_M1_p11, ML_ST_M1_2_4_5_7_p7, NC_ST_M1_2_3_5_6_p11, and EF_ST_M1_2_3_4_5_7_p10. LASSO (LAS), LAR, and stepwise (ST). M are models by fit criteria, p = #predictors selected.

For the best models presented in the main part of the article (Table 2, Table 3, Table 4, Table 5, Table 6 and Table 7), Venn diagrams are elaborated (Figure 14, Figure 15 and Figure 16). For *S. aureus*, the common variables occurring in all LAR, LASSO, and stepwise models are PASS_anti-inflam and PASS_antitumor (Figure 14). The set of two common variables is marked as “2” in the middle of Figure 14. For *S. aureus*, the number of common variables for LASSO with 11 selected variables (SA_LAS_M1_p11; yellow circle in Figure 14) and LAR models with 11 variables (SA_LAR_M1_2_5_p11; blue circle in Figure 14) is equal to the sum of intersections counts 2 + 4 = 6. According to Table 2 and Table 3, these variables are MW, PASS_anti-inflam, PASS_antitumor, perc_N, interaction of melting point with R2_substituent (4-nitrophenyl), and melting point squared. From the above variables, the signs, which indicate the direction of impact on MIC, are concordant for MW, PASS_antitumor (negative sign), PASS_anti-inflam, perc_N, and interaction of melting point with R2_substituent 4-nitrophenyl (positive sign). Adding a smaller stepwise selection model for *S. aureus* with only four variables, common variables occurring for the models LAR (M1, M2, M5; with 11 variables), LASSO (M1 with 11 variables), and stepwise (M5–M7 with 4 variables) are PASS_anti-inflam (positive sign) and PASS_antitumor (negative sign) (Figure 14, Table 2, Table 3 and Table 4).

After adding to LASSO (LAS), LAR, and stepwise (ST) for *S. aureus* (SA) the best models for *N. corallina* (NC) and *E. faecalis* (EF), we obtained the next Venn diagram (Figure 15). For wider set of models across four bacteria strains (including added models Stepwise (M1–M3) with 11 effects for *N. corallina* and Stepwise (M1–M5, M7) with 10 effects for *E. faecalis* (EF), one common variable is observed—PASS_anti-inflam (Table 2, Table 3, Table 4, Table 5 and Table 7). This one-variable intersection of selected sets of variables is marked as “1” in the middle of Figure 15.

MW (always with a negative sign) is the common variable chosen in models with at least seven effects selected, i.e., across four bacteria strains: *S. aureus* (SA), *N. corallina* (NC), *E. faecalis* (EF), and *M. luteus* (ML), as presented in Figure 16 (see Table 2, Table 3, Table 5, Table 6 and Table 7).

The presented tables (Table 2, Table 3, Table 4, Table 5, Table 6 and Table 7) indicate that SBC selects no more variables than AIC, and, most often, fewer. Similarly, the BIC criterion selects no more variables than AIC, and often fewer. This can be explained by the formulas in Section 4.4, which demonstrate that the BIC is more parsimonious, i.e., it penalizes models for free parameters in a more restricted way [23].

The average squared error validation (Val ASE) method makes it possible to assess the validity of effects (parameters) obtained on a random subset (70 percent of all tested chemicals) on the remaining 30 percent of the data, which replaces testing on a new independent sample. This validation (Val ASE) was performed and the results are presented as model 7 in Table 2 and Table 3 (or model 8, in the case of the stepwise method in Table 4, Table 5, Table 6 and Table 7). Moreover, the values of the adjusted coefficient of determination for the validation sample are usually similar to models based on all compounds.

Additionally, to maximize the use of the dataset, a 1000-fold bootstrap sampling of compounds was used to assess the frequency of variable selections in the models. These choice frequencies largely confirm the validity of the parameters. The high rate of selection of a variable into the model estimated using the bootstrap method is often confirmed by the high absolute value of the standardized coefficient (e.g., meltingTemp*R2_substituent_4-nitrophenyl in Table 2, RmoExper in Table 6, RmoExper and MW in Table 7).

In the interpretation of the impact of selected variables on MIC, assuming the other variables are not changed, negative values of coefficients indicate a decrease in MIC, while positive values indicate an increase (see Equations (1) and (2)). The impact of individual variables on the antibacterial activity of compounds depends on bacterial strains. Variables are considered important when they achieve the highest positive or smallest negative coefficients. Assuming that the remaining variables are constant, the following influence of variables on the antibacterial activity of compounds can be observed in followed models:

For LASSO models M1–M6 of *S. aureus* (Table 2, Figure 2), the results are as follows:

1. PASS_antitumor: an increase in this variable lowers the MIC value.
2. PASS_anti-inflam: as this variable increases, the MIC value increases.
3. MW: as the molar weight increases, the MIC value decreases.
4. melting Temp*R2_Substituent: assuming a constant melting point, the influence of different R2 substituents can be arranged in the order of having a favorable effect on the MIC value as follows: 2-pyridyl > 4-methylphenyl > 4-nitrophenyl.
5. MeltingTemp: an increase in this parameter increases the MIC value for all kinds of R1 and R2 substituents.

For LAR models M1–M5 and M7 of *S. aureus* (Table 3, Figure 4), the results are as follows:

1. PASS_antitumor: an increase in this variable lowers the MIC value.
2. R2_substituent 4-nitrophenyl: assuming a constant melting temperature, the presence of 4-nitrophenyl substituent in R2 increases the MIC value.
3. MeltingTemp: after summing the effect of meltingTemp, meltingTemp squared, and interaction with R2 substituent, an increase in meltingTemp causes an increase in the MIC value, which is bigger for 4-nitrophenyl than for the remaining for the remaining kinds of R2 substituents.
4. PASS_anti-inflam: as this variable increases, the MIC value increases.
5. PASS_antieczematic: as this variable increases, the MIC value increases.
6. MW: as the molar weight increases, the MIC value decreases.

For stepwise models M1–5 and M7 for *S. aureus* (Table 4, Figure 6), the results are as follows:

1. PASS_antitumor: as this variable increases, the MIC value decreases.
2. PASS_anti-inflam: as this variable increases, the MIC value increases.
3. perc_C: an increase in the percentage of carbon in a compound molecule causes a decrease in the MIC value.
4. miLOGP: lowering the lipophilicity value results in lower MIC values.

The importance of PASS_antitumor and PASS_anti-inflam with the described direction of impact on MIC in the M1–M5 and M7 models are confirmed in the validation ASE model, with a very close value of Adj R^2^ (41.15).

For the stepwise models M1–M3 and M5–M6 for *N. corallina* (Table 5, Figure 8), the results are as follows:

1. MW: as the molar weight increases, the MIC value decreases.
2. MeltingTemp*R2_substitituent: assuming the same melting point, the influence of different R2 substituents can be arranged in the order of having a beneficial effect on the MIC value as follows: 4-methylphenyl > 4-nitrophenyl > 2-pyridyl > phenyl.
3. The miLOGP variable has a greater impact on the MIC value than the experimental RmoExper; as the values of the two variables describing lipophilicity increase, the MIC value increases.
4. PASS_anti-inflam, PASS_antitumor, and PASS_antieczematic: as the value of each of these variables increases, the MIC value decreases.

For the stepwise models M1–M2, M4, and M7 for *M. luteus* (Table 6, Figure 10), the results are as follows:

1. RmoExper: as the lipophilicity increases, the MIC value increases.
2. RmoExper: as the molar weight increases, the MIC value decreases.
3. PASS_antieczematic: increasing the value of this variable causes a decrease in the MIC value.
4. PASS_antituberculosi: lowering the value of this variable lowers the MIC value.
5. MeltingTemp: as this variable increases, the MIC value decreases.

For the stepwise models M1–M5 and M7 of *E. faecalis* (Table 7, Figure 12), the results are as follows:

1. MW: as the molar weight increases, the MIC value decreases.
2. RmoExper: the lower the RmoExper value, the lower the MIC value.
3. Based on the interaction of the melting point and R1 substituent, and considering that the melting point does not change, the effect of R1 on the increase in MIC can be presented in descending order as 4-pyridyl > phenyl > 2-pyridyl. All substitutions increase the MIC, but the first one, 4-pyridyl, increases it the least and is the most favorable for this model. The weakest antibacterial activity is expected for the R1 substitution with 2-pyridyl.
4. PASS_anti-inflam: as this variable increases, the MIC value decreases.
5. PASS_antituberculosi: as this variable increases, the MIC value increases.
6. PASS_antibact: assuming other variables are not changed, the change in PASS_antibact by one SD increase the MIC value in 0.268199 (2.242303 − 1.974104), a lower value of this variable is more favorable.

Using the data collected in Table S26, the selected variables set as common for the modeling of the four bacterial strains *S. aureus*, *M. luteus*, *N. corallina*, and *E. faecalis* are as follows:

1. MW: as this variable increases, the MIC value decreases.
2. PASS_anti-inflam: as this variable increases, the MIC value decreases (except for *S. aureus*).
3. PASS_antieczematic: as this variable increases, the MIC value decreases (except for *S. aureus*).
4. MeltingTemp squared: in general, an increase in this variable causes a decrease in the MIC value (except for *E. faecalis*).
5. RmoExper: as lipophilicity decreases, the MIC value decreases.
6. PASS_antitumor: as this variable increases, the MIC value decreases.

In the majority of models, a beneficial effect of the increase in molar mass on the antibacterial activity was observed. However, it can be expected that this relationship will persist only up to a certain point, similar to previous studies on the relationship between the mass of chitosan derivatives and activity against *S. aureus* [35]. The compounds used for modeling had molar masses ranging from 291 to 411 g/mol.

The high frequency of selecting the PASS_anti-inflam and PASS_antieczem variables (Table S26) may seem surprising. However, the recently described potential use of anti-inflammatory drugs as antibacterial agents to combat biofilm formed by pathogenic bacteria [36] indicates the possibility of existing dependences between anti-inflammatory and antibacterial activity of the compounds. In turn, the selected PASS_antitumor variable may indicate the relationship between the cytotoxicity of compounds and antimicrobial activity. This may also confirm the possible potential of known anti-inflammatory and anti-cancer drugs, which may have antibacterial activity and are currently undergoing repositioning tests [37]. Interestingly, the PASS_antibact variable was selected less frequently, mainly in models involving *E. faecalis*.

The most frequently mentioned variables included the melting point in the second power (Table S26). The importance of the melting point in the analyzed models is also evidenced by its frequent presence in the interaction with R1 or R2 substituents. The influence of this variable on biological activity has not been widely studied so far, although several works on it have been published. The dependence of decomposition on the melting point of approved and withdrawn drugs was examined [38], as was the relationship between drug absorption and melting point [39]. The quantitative structure–property relationship, concerning the melting point of drug compounds, is another area of current research [40].

Lipophilicity plays a role not only in penetrating biological membranes but also in the metabolism, distribution, excretion, and toxicity of drugs [41]. It is worth noting that the experimental lipophilicity values (RmoExper) better explain the antibacterial activity than the calculated lipophilicity (miLOGP) values (only two selections in Table S26). This agrees with the suggestions of authors investigating this lipophilicity phenomenon using computational and chromatography methods [32,42].

Among other analyzed variables, the percentage composition of the tested compounds seems to play a secondary role, as the variables’ percentages of carbon and nitrogen in the composition of compounds were selected mainly in the models for *S. aureus*. Variables related to the number of hydrogen bond donors were selected more often than the number of hydrogen bond acceptors (Table S26), but their impact on MIC values, similarly to the topological polar surface area (TPSA) value, seems to be less important.

The variables R1 and R2 substituents, important from the point of view of the structure–activity relationship, occur as single main effects rather rarely, because interactions between substituents R1 or R2 and melting points are often present. For example, the stepwise model of *E. faecalis* predicts the highest antibacterial activity for the compounds possessing the R1 substituent 4-pyridyl and the lowest in the case of the 2-pyridyl substituent at the R1 position. Moreover, based on the stepwise model of *N. corallina*, the R2 substituents can be ranked from the most favorable in terms of antibacterial activity to the least favorable as follows: 4-methylphenyl > 4-nitrophenyl > 2-pyridyl > phenyl. However, LASSO and LAR models of *S. aureus* suggest that the R2 substituent 4-nitrophenyl is the least favorable for activity against this bacterial strain. These differences, however, may be caused by differences in individual bacterial strains.

More detailed studies of the influence of the above-mentioned variables, carried out on models built on a larger number of compounds with greater structural diversity, seem advisable to clarify the precise impact of these variables.

Generalized linear models for predicting the activity of chemical compounds from three groups (linear, 1,2,4-triazole derivatives, and cyclic imides), based on selected theoretical variables, lipophilicity, and the type of R1 and R2 substituents, with the interaction with the melting point, have beneficial predictive properties for the creation of compounds as potential drugs.

Despite obtaining significant results, it should be remembered that our work has certain limitations. Our models only concern antibacterial activity against five strains of Gram-positive bacteria. Only amidrazone derivatives from three groups (Figure 1b–d) were included in the analyzed data. Additionally, only R1 and R2 substituents with three and four categories, respectively, were included in the set of potential variables, additionally with the analysis of substituents’ interactions with the melting point. We intend to include other compounds to elaborate more general models. A higher number of agents will increase the size of the dataset, which may also let us analyze R3 and R4 substituents with a larger number of categories. We also plan to expand the analysis to a broader range of bacterial strains (including Gram-negative strains).

The classic approach to the design of antibacterial substances mainly considered the influence of substituents such as R1 and R2. Our approach to the design of antibacterial drugs extends the analysis to include the impact of other factors that may also affect the MIC value. Our models are multidimensional and also consider interactions between melting point and the R1 and R2 substituents.

For designing compounds with a similar chemical structure (i.e., amidrazone derivatives) for particular types of Gram-positive bacteria, we suggest using the variables that we obtained in our selection for single models and validated based on the ASE, or the variables that were most frequently selected based on the analysis of 1000 random samples using the bootstrap method.

## 4. Materials and Methods

### 4.1. Bacterial Strains

The antibacterial activity of compounds was tested against Gram-positive bacteria derived from the American Type Culture Collection (ATCC), including *Staphylococcus aureus* ATCC 25923 and *Enterococcus faecalis* ATCC 29,212, and from the Department of Genetic and Microbiology’s collection of Maria Curie Sklodowska University, including *Micrococcus luteus*, *Nocardia corallina* (currently *Gordonia rubripertincta*), and *Mycobacterium smegmatis*.

### 4.2. Determination of Minimum Inhibitory Concentration (MIC) In Vitro

The test was performed using the microdilution method in sterile 96-well microplates, following the procedure described by the Clinical and Laboratory Standards Institute [43]. All tested compounds were dissolved in dimethyl sulfoxide (DMSO) to obtain a concentration of 10.24 mg/mL and then were ten-fold diluted in Mueller–Hinton (MH) broth, followed by serial two-fold dilution in MH, resulting in a concentration range from 512 to 0.5 μg/mL. Each well was then inoculated with a bacterial suspension (0.5 McFarland standard). The plates were incubated at 37 °C for 18 h. Bacterial growth was confirmed spectrophotometrically using a microplate reader ASYS UVM 340 (Biogenet, Józefów, Poland). The MIC value was considered to be the lowest dilution of the compound that inhibited bacterial growth.

### 4.3. Predicted and Potential Predictive Variables in the Model

Models were evaluated for the antibacterial activity of the following five Gram-positive bacteria: *S. aureus* (n = 83), *M. smegmatis* (n = 85), *N. corallina* (n = 50), *E. faecalis* (n = 56), and *M. luteus* (n = 56). Experimentally identified minimal inhibitory concentrations (MICs) were the explained variables in the generalized linear models (GLMs) [25].

Eighty-five examined chemical compounds were obtained in the reaction of *N*^3^-substituted amidrazones with cyclic anhydrides, following methods described previously [12,13,14]. Generally, they belong to three groups depending on chemical structure (linear, 1,2,4-triazole derivatives, and cyclic imides, as shown in Figure 1). Individual compounds differ in the substituents R1 and R2, which are the only qualitative variables in the models. The structures of the studied compounds are presented in Figures S1–S3.

Among the 85 compounds with experimentally measured biological activities, we analyzed physicochemical properties such as molecular weight (MW), the per cent composition of oxygen, nitrogen, and carbon (calculated using the free ChemSketch software version 2017.1.2 Advanced Chemistry Development, Toronto, Ontario, Canada) [33]. Moreover, computational lipophilicity (miLOGP), the number of hydrogen NH and OH donors (Donors_H), the number of nitrogen and oxygen atoms (Acceptors_H), and the topological polar surface area (TPSA), values of the studied compounds were obtained using Molinspiration cheminformatics software [28].

Also, three categorial R1 substituents (2-pyridyl, 4-pyridyl, and phenyl) and four categorial R2 substitutions (2-pyridyl, 4-methylphenyl, 4-nitrophenyl, and phenyl) were included into set of proposed theoretical variables describing the compound. Potential biological actions (e.g., antibacterial, antituberculotic, anti-inflammatory, antitumor, and antieczematic) of the tested compounds were theoretically calculated using the PASS program (prediction for activity spectra for substances) [29].

Additionally, two experimental variables, such as experimental lipophilicity (R_M0,_ determined by thin layer chromatography using reversed-phased plates (nano-silica gel RP-18W, Fluca Analytical, Neu-Ulm, Germany) and 40–60% water–methanol mixtures) according to the method described previously [32] and a medium melting point (determined by MEL-Temp apparatus, Electrothermal, Stone, UK) were included into consideration. Interactions between melting temperature and R1, and also between melting temperature and R2 substituents, were also considered. Adding an interaction term to a MIC model becomes necessary when the statistical association between MIC and melting temperature depends on the value/level of the R1 or R2 substituent. Additionally, PASS antibacterial activity squared was considered. We anticipated that, among the five results from the PASS program, the PASS_antibact variable might be most closely related to MIC. To avoid duplicating variables, we decided to investigate this variable’s potential non-linear relationship by including its square in the set of potential variables. Abbreviations with descriptions of the potential explaining variables are given in Table 1.

Statistical generalized linear models (GLMs) estimate the influence of predictors on the outcome MIC. Most predictors are continuous, with two categorical variables: R1 and R2 substituents (Table 1). However, we also consider the modification of the impact of melting temperature on MIC by R1 substituent and the modification of the effect of melting temperature on MIC by R2 substituent. R1 and R2 may be regarded as moderating variables. If this effect modification exists, it is a statistical interaction. For example, we may model the impact of melting temperature on MIC that is modified by three different R1 substituents (1 = 2-pyridyl; 2 = 4-pyridyl; 3 = phenyl) or by R2 substituent (1 = 2-pyridyl; 2 = 4-methylphenyl; 3 = 4-nitrophenyl phenyl). Interaction variables are generated by multiplying melting temperature by the level of R1 (or R2), and this resulting product variable is then entered into the model as an additional potential predictor, along with the melting temperature and R1 (R2).

### 4.4. Variables Selection for Generalized Linear Models

For multidimensional problems, dimension reduction to represent the problem without losing significant amounts of information is crucial. The selection of variables (main effects or interaction effects) was performed using three types of generalized linear model (GLM) selection models: adaptive LASSO, least-angle regression (LAR), and stepwise selection. The maximal number of variables built into the model was bounded by a value depending on the number of training examples for MIC: for n > 80 it was set to 13 (for *S. aureus* n = 83, *M. smegmatis* n = 85), and for others, it was set to 10 (*N. corallina* n = 50, *M. luteus* n = 56, and *E. faecalis* n = 56).

LASSO selection (least absolute shrinkage and selection operator for generalized linear models) [25] is a regularization technique to reduce the number of predictors in a generalized linear model. LASSO is used to identify important predictors and to choose among redundant ones. The LASSO method is a penalized method that considers all candidate variables and reduces the coefficients of non-important variables to zero. As the penalty term increases, the LASSO technique sets more coefficients to zero, resulting in a smaller model with fewer predictors. By making shrinkage estimates, LASSO potentially could result in lower predictive errors than ordinary least squares. Adaptive LASSO is a method for regularization and variable selection in regression analysis that was introduced by Zou [44]. Zou demonstrated that the adaptive LASSO has theoretical advantages over the standard LASSO. It is beneficial when the explaining variables are correlated and there is a need to select a small subset of important predictors for the model. By introducing weights (a prior “intelligence” about variables), adaptive LASSO is likely to penalize non-zero coefficients less than the zero ones. In GLMs, variable selection using LASSO is often applied [45].

Least-angle regression (LAR) was introduced by Efron et al. [46]. The LAR model-selection method is based on traditional forward selection, where, given a collection of possible explanatory variables, the one having the largest absolute correlation with the explained variable is selected.

Stepwise (forward–backwards) model selection is a classical model selection and dimensionality reduction technique. The same entry and removal significance levels for the forward selection and backward elimination were used to evaluate the contributions of variables as they are added to or removed from a model. The entry significance level (SLE) was set to 0.15 and the stay significance level (SLS) was also set to 0.15.

Variables selection within the framework of general linear models was performed using the procedure of variables selection in GLM models (GLMSELECT) procedure in the SAS package [31,47]. A variety of fit statistics were specified as criteria for selection. Seven or eight criteria, such as Adj R^2^ (resulting model denoted as M1), AIC (M2), AICC (M3), BIC (M4), C(p) (M5), SBC (M6), PRESS (M7—for stepwise selection only), and ASE (M7 or M8 for stepwise selection), were applied. The process of adding or removing variables was stopped according to the optimal values of performance statistics, where minimal values are considered optimal for all performance statistics except adjusted R^2^ (Table 8).

R^2^ determines the proportion of variance in the dependent variable that can be explained by the independent variable; hence, it is called the determination coefficient. It serves as a measure of goodness of fit for the model. The adjusted determination coefficient takes into account the number of predictors and is calculated as follows:

Adj R^2^ = 1 − (n − 1)(1 − R^2^)/(n − p), (3)

where n is the number of observations used to fit the model, p is the number of parameters (effects, including the intercept) in the model, and n is the number of observations.

The AIC and SBC are both methods of assessing model fit penalized for the number of estimated parameters. Akaike’s information criterion is defined as follows:

AIC = nxln(SSE/n) + 2p,(4)

where SSE is the sum of squared errors (residual sum of squares).

The corrected AIC is given by the following equation:

AICC = n log(SSE/n) + (n + p)/(1 − (p + 2)/n),(5)

Schwarz’s Bayesian criterion (BIC) is calculated as follows:

SBC = n log(SSE/n) + p log(n)(6)

Sawa’s Bayesian Information Criterion (BIC) additionally takes into account the pure error variance *σ*^2^ fitting the full model, as follows:

BIC = n log(SSE/n) + 2(p + 2)q − 2(q^2^), (7)

where q = n(s^2^)/SSE and s^2^ is the estimate of pure error variance *σ*^2^ from fitting the full model.

Mallows’ statistic is defined as follows:

C(p)= SSE/*s*^2^ + 2p − n. (8)

The smallest values of AIC, AICC, SBC, C(p), and BIC are preferred.

To compare the results of creating single models with resampling of data models, (where model selection is repeated on subsets of the input data and parameters are averaged), the method of model averaging (MODELAVERAGE) statement in GLMSELECT procedure from the SAS package [31,47] was applied. Resampling-based methods, in which samples are obtained by drawing with replacements, called bootstrap, were used. Bootstrap in the context of variable selection was proposed by Breiman [48]. A thousand replications were used in our bootstrap samples.

## 5. Conclusions

Using GLMs, statistically significant models were obtained for the Gram-positive bacterial strains. The best results based on the optimum adjusted determination coefficient and other fit criteria, e.g., Akaike’s information criterion, were obtained for *S. aureus*, indicating an opportunity to support the design of antibacterial drugs using this method and selected predictors. Variables with the greatest impact on compound activity in inhibiting *S. aureus* growth included PASS_antitumor, PASS_anti-inflam, and the interaction of the melting point with the R2 substituent (4-nitrophenyl). The weakest models with an adjusted determination coefficient below 20% were obtained for the *M. smegmatis* strain, which may be due to the different properties of the mycobacterial cells.

For all bacterial strains, the most often selected main effects in models were molecular weight, PASS_antieczematic, PASS_anti-inflam, PASS_antitumor, experimental RmoExper lipophilicity, and squared melting temperature. For several models, the influence of R1 and R2 substituents on the antibacterial activity against a given bacterial strain was determined. For example, the stepwise model predicted the highest antibacterial activity for the compounds possessing the R1 substituent 4-pyridyl and the lowest in the case of the 2-pyridyl substituent at the R1 position against *E. faecalis*. Another stepwise model for *N. corallina* suggested the beneficial influence of substituents on the MIC value in the following order: 4-methylphenyl > 4-nitrophenyl > 2-pyridyl > phenyl. The selection of the interaction of melting point with substituents R1 and R2 indicates that the relationship with MIC depends on the substituent type. The models found can constitute the basis for building further strategies that may be useful in designing new antibacterial drugs.

## Figures and Tables

**Table 1:** Potential variables in models.

	Abbreviation	Description	Data Source
1	MW	molecular weight	Molinspiration [28]
2	RmoExper	lipophilicity index (experimental lipophilicity)	experimental [32]
3	miLOGP	theoretical lipophilicity	Molinspiration [28]
4	PASS_antibact	antibacterial activity calculated using PASS program	PASS [29]
5	PASS_anti*PASS_antib	PASS antibacterial activity squared	SAS [31]
6	PASS_anti-inflam	anti-inflammatory activity calculated using PASS program	PASS [29]
7	PASS_antieczematic	antieczematic activity calculated using PASS program	PASS [29]
8	PASS_antitumor	antitumor activity calculated using PASS program	PASS [29]
9	PASS_antituberculosi	antituberculotic activity calculated using PASS program	PASS [29]
10	perc_O	percent of oxygen	ChemSketch [33]
11	perc_N	percent of nitrogen	ChemSketch [33]
12	perc_C	percent of carbon	ChemSketch [33]
13	Donors_H	donors H	Molinspiration [28]
14	Acceptors_H	acceptors H	Molinspiration [28]
15	TPSA	topological polar surface area	Molinspiration [28]
16	R1_substituent	substituent R1 (2-pyridyl, 4-pyridyl, phenyl)	Figures S1–S3
17	R2_substituent	substituent R2 (2-pyridyl, 4-methylphenyl, 4-nitrophenyl, phenyl)	Figures S1–S3
18	meltingTemp*R1_substituent	interaction term of meltingTemp with R1_substituent	SAS [31]
19	meltingTemp*R2_substituent	interaction term of meltingTemp with R2_substituent	SAS [31]
20	meltingTemp	melting temperature	experimental
21	meltingTe*meltingTem	melting temperature squared	SAS [31]

**Table 2:** *S. aureus* parameter estimates for LASSO models optimized by the seven fit criteria.

Parameter	Estimate b_i_			Standardized Estimate β_i_			Mean Selection % from Bootstrap
	Model 1, 2, and 5 ****	Model 3, 4, and 6 ****	Model 7 ***	Model 1, 2, and 5 ****	Model 3, 4, and 6 ****	Model 7 ***	
Intercept	873.169475	1114.556967	896.693393	0	0	0	
MW	−2.046303	−2.012174		−0.174756	−0.171842		61.51
PASS_anti-inflam	617.458702	340.124433	615.083438	0.325141	0.179103	0.327629	56.96
PASS_antitumor	−1243.082270	−981.273647	−1179.549012	−0.391309	−0.308895	−0.356949	82.10
perc_N	4.769786		8.932653	0.040680		0.071365	30.81
TPSA	2.715861			0.178460			40.87
R2_substituent_4-nitrophenyl	−658.793078			−0.648931			39.80
meltingTemp*R2_substituent_2-pyridyl	−0.339383	−0.161304	−0.022462	−0.073312	−0.034844	−0.004726	52.94
meltingTemp*R2_substituent_4-methylphenyl	0.401398	0.394636	0.433419	0.094897	0.093298	0.088599	67.59
meltingTemp*R2_substituent_4-nitrophenyl	5.050186	1.760136	1.068188	0.862991	0.300777	0.170470	86.96
meltingTemp*R1_substituent_2-pyridyl	−0.234074		−0.554738	−0.064224		−0.150870	44.10
meltingTe*meltingTem	0.001853	0.002906	0.001773	0.090542	0.142001	0.091189	75.87
PASS_antieczematic			14.583163			0.010580	21.26
perc_C			−4.911495			−0.059439	18.14
Acceptors_H			−30.086571			−0.105408	36.66
R1_substituent_4-pyridyl			41.829359			0.051032	17.94
R^2^	0.5160	0.4542	0.4983	0.5160	0.4542	0.4983	
Adj R^2^	0.4410	0.4032	0.3645	0.4410	0.4032	0.3645	
#effects	12	8	13	11	7	12	

Model 1 by Adj R^2^, Model 2 by AIC, Model 3 by AICC, Model 4 by BIC, Model 5 by C(p), Model 6 by SBC, and Model 7 by average squared error validation (ASE Val); **** *p* < 0.0001; *** *p* = 0.0006.

**Table 3:** *S. aureus* parameter estimates for LAR models optimized by seven fit criteria.

Parameter	Estimate b_i_				Standardized Estimate β_i_				Mean Selection % from Bootstrap
	Model 1 ****	Model 2, 3, 4, and 5 ****	Model 6 ****	Model 7 ***	Model 1 ****	Model 2, 3, 4, and 5 ****	Model 6 ****	Model 7 ***	
Intercept	462.898124	462.505125	583.823298	381.403651	0	0	0	0	
MW	−1.069639	−0.568965		−0.259356	−0.091348	−0.048590		−0.021098	86.01
PASS_anti-inflam	463.251051	411.429060	316.932193	470.435188	0.243939	0.216650	0.166890	0.237494	23.13
PASS_antieczematic	163.963667	148.389990		295.282144	0.127085	0.115014		0.213817	33.73
PASS_antitumor	−1369.627613	−1267.557881	−926.310045	−1199.879404	−0.431145	−0.399014	−0.291593	−0.384189	52.26
PASS_antituberculosi	199.819895	158.913470		7.935571	0.081416	0.064749		0.003424	37.16
perc_N	4.202615			10.150326	0.035843			0.088231	10.10
R2_substituent_4-methylphenyl	21.291074				0.026453				11.90
meltingTemp*R2_substituent_4-nitrophenyl	1.553781	1.088246		0.474492	0.265515	0.185963		0.071326	10.07
meltingTemp*R1_substituent_4-pyridyl	0.189909				0.045909				24.60
meltingTemp	3.393038	2.624292		0.178016	0.435941	0.337172		0.023650	65.09
meltingTe*meltingTem	−0.008066	−0.006092			−0.394171	−0.297722			35.79
PASS_antibact				44.616304				0.012817	38.34
perc_C				−0.395393				−0.004806	9.99
R^2^	0.4978	0.4680	0.3131	0.4879	0.4978	0.4680	0.3131	0.4879	
Adj R^2^	0.4200	0.4104	0.2959	0.3715	0.4200	0.4104	0.2959	0.3715	
#effects	12	9	3	11	11	8	2	10	

Model 1 by Adj R^2^, Model 2 by AIC, Model 3 by AICC, Model 4 by BIC, Model 5 by C(p), Model 6 by SBC, Model 7 by ASE Val; **** *p* < 0.0001; *** *p* = 0.0004.

**Table 4:** *S. aureus* parameter estimates for stepwise models optimized by eight criteria.

Parameter	Estimate b_i_			Standardized Estimate β_i_			Mean Selection % from Bootstrap
	Model 1, 2, 3, 4, 5, and 7 ****	Model 6 ****	Model 8 ****	Model 1, 2, 3, 4, 5, and 7 ****	Model 6 ****	Model 8 ****	
Intercept	2429.212180	1853.690776	654.612436	0	0	0	
miLOGP	76.605993			0.205949			24.58
PASS_anti-inflam	732.638700	820.792894	689.123870	0.385793	0.432213	0.367067	29.94
PASS_antitumor	−1628.150512	−1633.785168	−1564.692751	−0.512525	−0.514299	−0.473499	19.70
perc_C	−29.306596	−18.583962		−0.374185	−0.237279		14.80
R^2^	0.4415	0.4256	0.4321	0.4415	0.4256	0.4321	
Adj R^2^	0.4129	0.4038	0.4115	0.4129	0.4038	0.4115	
#effects	5	4	3	4	3	2	

Model 1 by Adj R^2^, Model 2 by AIC, Model 3 by AICC, Model 4 by BIC, Model 5 by C(p), Model 6 by SBC, Model 7 by PRESS, Model 8 by ASE Val; **** *p* < 0.0001.

**Table 5:** *N. corallina* parameter estimates for stepwise models optimized by eight fit criteria.

Parameter	Estimate b_i_			Standardized Estimate β_i_			Mean Selection % from Bootstrap
	Model 1, 2, 3, 5, and 6 ****	Model 4 and 7 ****	Model 8 ***	Model 1, 2, 3, 5, and 6 ****	Model 4 and 7 ****	Model 8 ***	
Intercept	1390.328152	1291.954852	705.248202	0	0	0	
MW	−8.219504	−6.992417		−1.060433	−0.902121		42.46
RmoExper	89.254456	83.115214	111.265824	0.276959	0.257909	0.355463	39.91
miLOGP	138.390618	130.292793		0.638554	0.601189		14.90
PASS_anti-inflam	−836.304090	−758.846240		−0.626581	−0.568547		42.31
PASS_antieczematic	−251.261248	−384.382216	−364.349190	−0.293190	−0.448525	−0.444599	61.77
PASS_antitumor	−711.157471			−0.324943			17.99
meltingTe*R2_substit 2-pyridyl	20.988569	14.778770		7.312478	5.148966		41.97
meltingTe*R2_substit 4-methylphenyl	21.371754	15.514874		5.927923	4.303389		41.97
meltingTe*R2_substit 4-nitrophenyl	21.756328	15.380206		6.202207	4.384528		41.97
meltingTe*R2_substit phenyl	19.951998	14.272193		8.530327	6.101969		41.97
meltingTe*meltingTem	−0.060928	−0.042149		−3.875007	−2.680625		37.34
R^2^	0.6645	0.6316	0.3146	0.6645	0.6316	0.3146	
Adj R^2^	0.5674	0.5371	0.2730	0.5674	0.5371	0.2730	
#effects	12	11	3	11	10	2	

Model 1 by Adj R^2^, Model 2 by AIC, Model 3 by AICC, Model 4 by BIC, Model 5 by C(p), Model 6 by SBC, Model 7 by PRESS, Model 8 by ASE Val; **** *p* < 0.0001; *** *p* = 0.0020.

**Table 6:** *M. luteus* parameter estimates for stepwise models optimized by seven fit criteria.

Parameter	Estimate b_i_				Standardized Estimate β_i_				Mean Selection % from Bootstrap
	Model 1, 2, 4, 5, and 7 ****	Model 3 ****	Model 6 ****	Model 8 ***	Model 1, 2, 4, 5, and 7 ****	Model 3 ****	Model 6 ****	Model 8 ***	
Intercept	1140.676275	828.893739	−9.630019	705.248202	0	0	0	0	
MW	−3.248648	−2.773553			−0.356696	−0.304531			60.12
RmoExper	251.327244	234.240999	186.230637		0.682719	0.636305	0.505887		88.91
PASS_antieczematic	−224.221116	−245.759841			−0.231603	−0.253851			39.67
PASS_antituberculosi	404.111567	390.887022			0.206186	0.199439			41.54
meltingTemp	−1.067538				−0.173456				19.75
PASS_anti-inflam				−775.174645				−0.631067	25.45
Donors_H				−146.944623				−0.771423	36.64
R^2^	0.4149	0.3890	0.2559	0.3155	0.4149	0.3890	0.2559	0.3155	
Adj R^2^	0.3564	0.3410	0.2421	0.2795	0.3564	0.3410	0.2421	0.2795	
#effects	6	5	2	3	5	4	1	2	

Model 1 by Adj R^2^, Model 2 by AIC, Model 3 by AICC, Model 4 by BIC, Model 5 by C(p), Model 6 by SBC, Model 7 by PRESS, Model 8 by ASE Val; **** *p* < 0.0001; *** *p* = 0.0007.

**Table 7:** *E. faecalis* parameter estimates for stepwise models optimized by eight fit criteria.

Parameter	Estimates b_i_			Standardized Estimates β_i_			Mean Selection % from Bootstrap
	Model 1, 2, 3, 4, 5, and 7 ****	Model 6 ****	Model 8 ***	Model 1, 2, 3, 4, 5, and 7 ****	Model 6 ****	Model 8 ***	
Intercept	2131.035479	1766.168419	2399.703970	0	0	0	
MW	−7.857123	−5.743976	−6.011646	−0.645950	−0.472224	−0.492079	80.76
RmoExper	297.848654	263.084487		0.605812	0.535104		94.21
PASS_antibact	−6923.260504	−6688.826548		−1.974104	−1.907257		10.04
PASS_anti*PASS_antib	24935	23661		2.242303	2.127725		5.69
PASS_anti-inflam	−662.638878		−827.312567	−0.329342		−0.435166	10.26
PASS_antituberculosi	855.244926	865.830163		0.326730	0.330774		13.77
meltingTe*R1_substit 2-pyridyl	2.496836			0.653657			33.47
meltingTe*R1_substit 4-pyridyl	0.931634			0.203136			33.47
meltingTe*R1_substit phenyl	2.178632			0.490410			33.47
meltingTe*meltingTem			0.005607			0.267285	15.83
R^2^	0.5679	0.4266	0.2944	0.5679	0.4266	0.2944	
Adj R^2^	0.4834	0.3693	0.2372	0.4834	0.3693	0.2372	
#effects	10	6	4	9	5	3	

Model 1 by Adj R^2^, Model 2 by AIC, Model 3 by AICC, Model 4 by BIC, Model 5 by C(p), Model 6 by SBC, Model 7 by PRESS, Model 8 by ASE Val; **** *p* < 0.0001; *** *p* = 0.0045.

**Table 8:** Fit criteria applied and denotation of final models after achieving optimal fit value or after a pre-assumed maximal number of variables.

ID	Name	Meaning	LASSO	LAR	Stepwise
1	Adj R^2^	R^2^ statistic adjusted for degrees of freedom	M1	M1	M1
2	AIC	Akaike’s information criterion	M2	M2	M2
3	AICC	Corrected Akaike’s information criterion	M3	M3	M3
4	BIC	Sawa Bayesian information criterion	M4	M4	M4
5	CP	Mallows’ C(p) statistic	M5	M5	M5
6	SBC	Schwarz Bayesian information criterion	M6	M6	M6
7	PRESS	Predicted residual sum of squares			M7
8	VALIDATION ASE	Average square error for the validation data (30%)	M7	M7	M8

## Data Availability

Dataset available on request from the authors.

## Supplementary Materials

The following supporting information can be downloaded at: https://www.mdpi.com/article/10.3390/molecules29102369/s1, Figure S1: the structures of studied compounds 1–24. Figure S2: the structures of studied compounds 25–54; Figure S3: the structures of studied compounds 55–85. Table S1: *S. aureus*. LASSO models were selected according to the criteria. Fit statistics with F-values from ANOVA (n = 83). Table S2: *S. aureus*. LAR models were selected according to the criteria. Fit statistics with F-values from ANOVA (n = 83). Table S3: *S. aureus*. Stepwise models were selected according to the criteria. Fit statistics with F-values from ANOVA (n = 83). Table S4: *N. corallina*. LASSO models were selected according to criteria. Fit statistics with F-values from ANOVA (n = 50). Table S5: *N. corallina*. LAR models were selected according to the criteria. Fit statistics with F-values from ANOVA (n = 50). Table S6: *N. corallina*. Stepwise models were selected, according to criteria. Fit statistics with F-values from ANOVA (n = 50). Table S7: *M. luteus*. LASSO models were selected according to criteria. Fit statistics with F-values from ANOVA (n = 56). Table S8: *M. luteus*. LAR models were selected according to criteria. Fit statistics with F-values from ANOVA (n = 56). Table S9: *M. luteus*. Stepwise models were selected according to criteria. Fit statistics with F-values from ANOVA (n = 56). Table S10: *E. faecalis*. LASSO models were selected according to criteria. Fit statistics with F-values from ANOVA (n = 56). Table S11: *E. faecalis*. LAR models were selected according to criteria. Fit statistics with F-values from ANOVA (n = 56). Table S12: *E. faecalis*. Stepwise models were selected according to criteria. Fit statistics with F-values from ANOVA (n = 56). Table S13: *M. smegmatis*. LASSO models were selected according to criteria. Fit statistics with F-values from ANOVA (n = 85). Table S14: *M. smegmatis*. LAR models were selected according to criteria. Fit statistics with F-values from ANOVA (n = 85). Table S15: *M. smegmatis*. Stepwise models were selected according to criteria. Fit statistics with F-values from ANOVA (n = 85). Table S16: *N. corallina*. Parameter estimates for LASSO models were optimized by seven fit criteria. Table S17: *N. corallina*. Parameter estimates for LAR models were optimized by seven fit criteria. Table S18: *M. luteus*. Parameter estimates for LASSO models were optimized by seven fit criteria. Table S19: *M. luteus*. Parameter estimates for LAR models were optimized by seven fit criteria. Table S20: *E. faecalis*. Parameter estimates for LASSO models were optimized by seven fit criteria. Table S21: *E. faecalis*. Parameter estimates for LAR models were optimized by seven fit criteria. Table S22: *M. smegmatis*. Parameter estimates for LASSO models were optimized by seven fit criteria. Table S23: *M. smegmatis*. Parameter estimates for Stepwise models were optimized by eight fit criteria. Table S25: Presence of main effects with positive and negative signs in Table 1, Table 2, Table 3, Table 4, Table 5, Table 6 and Tables S16–S24 for all considered bacterial strains *S. aureus* (SA), *M. luteus* (ML), *N. corallina* (NC), *E. faecalis* (EF), and *M. smegmatis* (MS). Table S26: Presence of main effects with positive and negative signs in Table 1, Table 2, Table 3, Table 4, Table 5, Table 6 and Tables S16–S21 for four bacterial strains *S. aureus* (SA), *M. luteus* (ML), *N. corallina* (NC), and *E. faecalis* (EF).
