# Supplementary material for: Modeling of Effectiveness of N3-Substituted Amidrazone Derivatives as Potential Agents against Gram-Positive Bacteria
Source: Molecules. 2024 May 17;29(10):2369. doi: 10.3390/molecules29102369 (PMC11124365; doi:10.3390/molecules29102369)
Supplement: Supplementary file 1 [file molecules-29-02369-s001.zip › molecules-2978653-supplementary.pdf]

Modeling of Effectiveness of N^3-Substituted Amidrazone Derivatives as Potential Agents against Gram-Positive Bacteria

Małgorzata Ćwiklińska-Jurkowska [1], Renata Paprocka [2,*], Godwin Munroe Mwaura [3] and Jolanta Kutkowska [4]

[1] Department of Biostatistics and Theory of Biomedical Systems, Faculty of Pharmacy, Collegium Medicum in Bydgoszcz, Nicolaus Copernicus University in Toruń, Jagiellońska Str. 15, 85-067 Bydgoszcz, Poland; mjurkowska@cm.umk.pl

[2] Department of Organic Chemistry, Faculty of Pharmacy, Collegium Medicum in Bydgoszcz, Nicolaus Copernicus University in Toruń, Jurasza Str. 2, 85-089 Bydgoszcz, Poland

[3] Department of Pharmaceutical Chemistry, Pharmaceutics and Pharmacognosy, Faculty of Health Sciences, University of Nairobi, KNH, Nairobi P.O. Box 2149-00202, Kenya; godwinmunroe1@gmail.com

[4] Department of Genetics and Microbiology, Institute of Biological Sciences, Maria Curie-Skłodowska University, Akademicka Str. 19, 20-033 Lublin, Poland; jolanta.kutkowska@mail.umcs.pl

* Correspondence: renata.bursa@cm.umk.pl

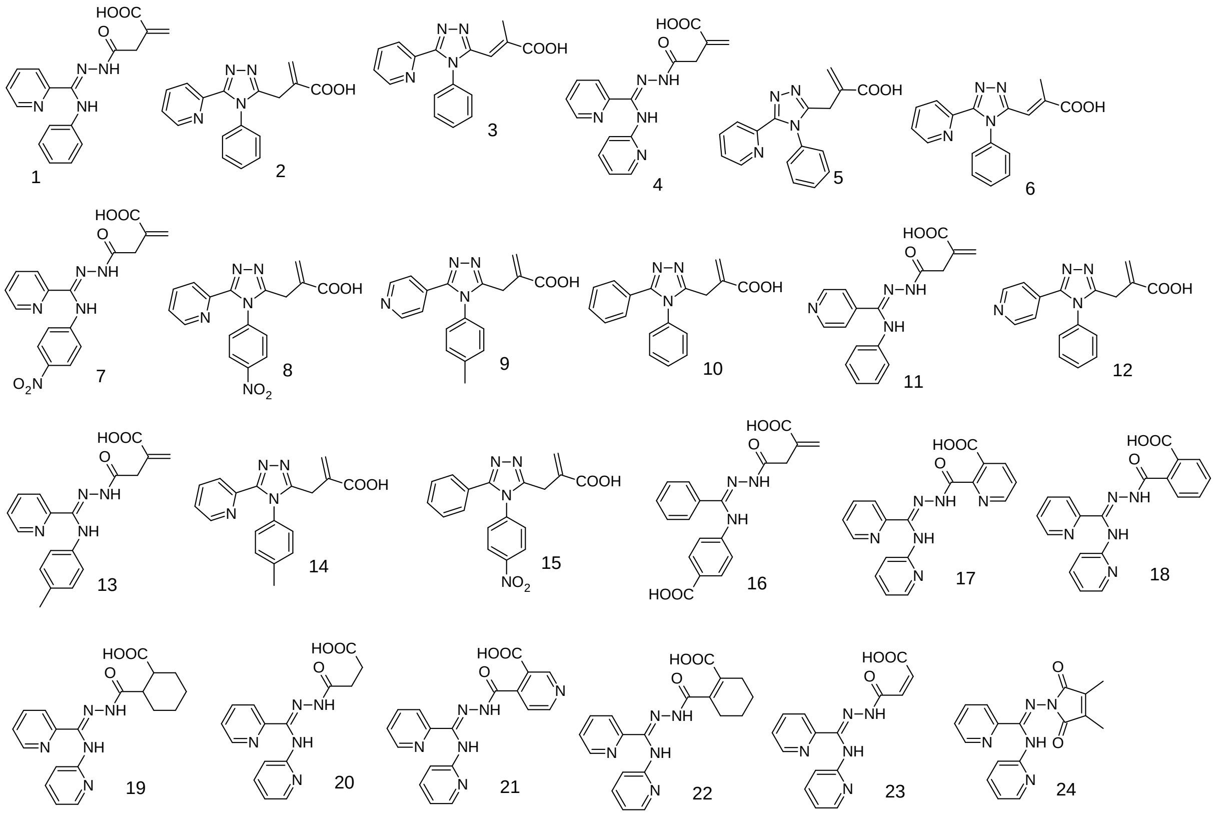

Figure S1. The structures of studied compounds 1-24.

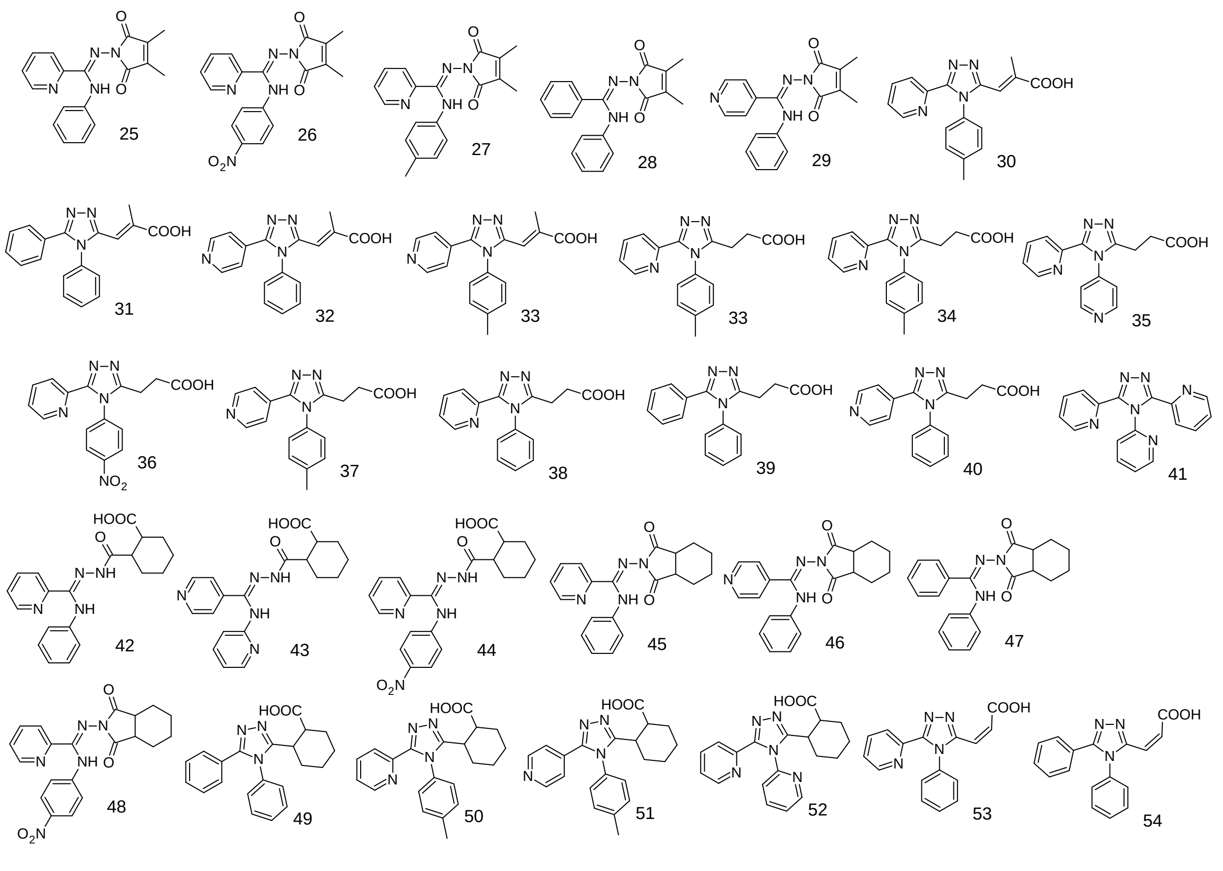

Figure S2. The structures of studied compounds 25-54

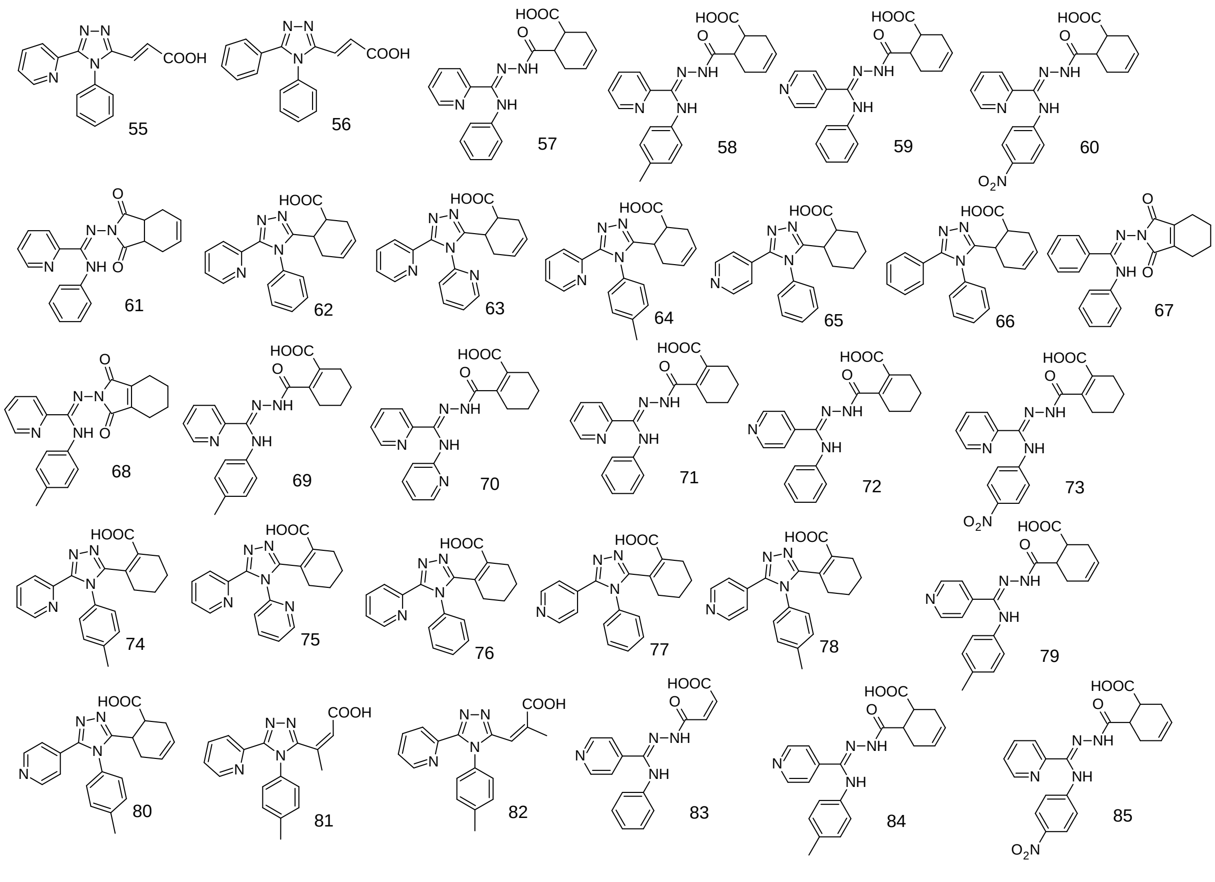

Figure S3. The structures of studied compounds 55-85

SMILES notation of compounds:

1) O=C(O)C(=C)CC(=O)N\N=C(/Nc1ccccc1)c1ccccn1
2) O=C(O)C(=C)Cc1nnc(c2ccccn2)n1c1ccccc1
3) O=C(O)C(\C)=C\c1nnc(c2ccccn2)n1c1ccccc1
4) O=C(O)C(=C)CC(=O)N\N=C(/Nc1ccccn1)c1ccccn1
5) O=C(O)C(=C)Cc1nnc(c2ccccn2)n1c1ccccc1
6) O=C(O)C(\C)=C\c1nnc(c2ccccn2)n1c1ccccc1
7) O=C(O)C(=C)CC(=O)N\N=C(/Nc1ccc(cc1)[N+]([O-])=O)c1ccccn1
8) [O-][N+](=O)c1ccc(cc1)n1c(nnc1CC(=C)C(=O)O)c1ccccn1
9) O=C(O)C(=C)Cc1nnc(c2ccncc2)n1c1ccc(C)cc1
10) O=C(O)C(=C)Cc1nnc(c2ccccc2)n1c1ccccc1
11) O=C(O)C(=C)CC(=O)N\N=C(/Nc1ccccc1)c1ccncc1
12) O=C(O)C(=C)Cc1nnc(c2ccncc2)n1c1ccccc1
13) O=C(O)C(=C)CC(=O)N\N=C(/Nc1ccc(C)cc1)c1ccccn1
14) O=C(O)C(=C)Cc1nnc(c2ccccn2)n1c1ccc(C)cc1
15) C1=CC=CC=C1C2=NN=C(CC(=C)C(O)=O)N2C3=CC=C(N(=O)=O)C=C3
16) O=C(O)C(=C)CC(=O)N\N=C(/Nc1ccc(cc1)[N+]([O-])=O)c1ccccc1
17) O=C(O)c1cccnc1C(=O)N\N=C(/Nc1ccccn1)c1ccccn1
18) O=C(O)c1ccccc1C(=O)N\N=C(\Nc1ccccn1)c1ccccn1
19) O=C(O)[C@@H]1CCCC[C@@H]1C(=O)N\N=C(/Nc1ccccn1)c1ccccn1
20) O=C(O)CCC(=O)N\N=C(/Nc1ccccn1)c1ccccn1
21) O=C(O)c1cnccc1C(=O)N\N=C(/Nc1ccccn1)c1ccccn1
22) O=C(O)C1CC=CCC1C(=O)N\N=C(/Nc1ccccn1)c1ccccn1
23) O=C(O)/C=C\C(=O)N\N=C(/Nc1ccccn1)c1ccccn1
24) O=C1C(C)=C(C)C(=O)N1/N=C(\Nc1ccccn1)c1ccccn1
25) O=C1C(C)=C(C)C(=O)N1/N=C(\Nc1ccccc1)c1ccccn1
26) O=N(=O)c1ccc(N\C(=N/N2C(=O)C(C)=C(C)C2=O)c2ccccn2)cc1
27) Cc1ccc(N\C(=N/N2C(=O)C(C)=C(C)C2=O)c2ccccn2)cc1
28) O=C1C(C)=C(C)C(=O)N1/N=C(\Nc1ccccc1)c1ccccc1
29) O=C1C(C)=C(C)C(=O)N1/N=C(\Nc1ccccc1)c1ccncc1
30) O=C(O)C(\C)=C\c1nnc(c2ccccn2)n1c1ccc(C)cc1
31) O=C(O)C(\C)=C\c1nnc(c2ccccc2)n1c1ccccc1
32) O=C(O)C(\C)=C\c1nnc(c2ccncc2)n1c1ccccc1
33) O=C(O)C(\C)=C\c1nnc(c2ccncc2)n1c1ccc(C)cc1
34) O=C(O)CCc1nnc(c2ccccn2)n1c1ccc(C)cc1
35) O=C(O)CCc1nnc(c2ccccn2)n1c1ncccc1
36) [O-][N+](=O)c1ccc(cc1)n1c(nnc1CCC(=O)O)c1ccccn1
37) O=C(O)CCc1nnc(c2ccncc2)n1c1ccc(C)cc1
38) O=C(O)CCc1nnc(c2ccccn2)n1c1ccccc1
39) O=C(O)CCc1nnc(c2ccccc2)n1c1ccccc1
40) O=C(O)CCc1nnc(c2ccncc2)n1c1ccccc1
41) c1cnc(cc1)n1c(nnc1c1ccccn1)c1ccccn1
42) O=C(O)C1CCCCC1C(=O)N\N=C(/Nc1ccccc1)c1ccccn1
43) O=C(O)C1CCCCC1C(=O)N\N=C(/Nc1ccccc1)c1ccncc1
44) O=C(O)C1CCCCC1C(=O)N\N=C(/Nc1ccc(cc1)[N+]([O-])=O)c1ccccn1
45) O=C1C2CCCCC2C(=O)N1/N=C(/Nc1ccccc1)c1ccccn1
46) O=C1C2CCCCC2C(=O)N1/N=C(/Nc1ccccc1)c1ccncc1

47) O=C1C2CCCCC2C(=O)N1/N=C(/Nc1ccccc1)c1ccccc1
48) [O-][N+](=O)c1ccc(cc1)N/C(=N/N1C(=O)C2CCCCC2C1=O)c1ccccc1
49) O=C(O)C1CCCCC1c1nnc(c2ccccc2)n1c1ccccc1
50) O=C(O)C1CCCCC1c1nnc(c2ccccn2)n1c1ccc(C)cc1
51) O=C(O)C1CCCCC1c1nnc(c2ccncc2)n1c1ccc(C)cc1
52) O=C(O)C1CCCCC1c1nnc(c2ccccn2)n1c1ncccc1
53) O=C(O)/C=C\c1nnc(c2ccccn2)n1c1ccccc1
54) O=C(O)/C=C\c1nnc(c2ccccc2)n1c1ccccc1
55) O=C(O)/C=C/c1nnc(c2ccccn2)n1c1ccccc1
56) O=C(O)/C=C/c1nnc(c2ccccc2)n1c1ccccc1
57) O=C(O)C1CC=CCC1C(=O)N\N=C(/Nc1ccccc1)c1ccccn1
58) O=C(O)C1CC=CCC1C(=O)N\N=C(/Nc1ccc(C)cc1)c1ccccn1
59) O=C(O)C1CC=CCC1C(=O)N\N=C(/Nc1ccccc1)c1ccncc1
60) O=C(O)C1CC=CCC1C(=O)N\N=C(/Nc1ccc(cc1)[N+]([O-])=O)c1ccncc1
61) O=C1C2CC=CCC2C(=O)N1/N=C(\Nc1ccccc1)c1ccccn1
62) O=C(O)C1CC=CCC1c1nnc(c2ccccn2)n1c1ccccc1
63) O=C(O)C1CC=CCC1c1nnc(c2ccccn2)n1c1ncccc1
64) O=C(O)C1CC=CCC1c1nnc(c2ccccn2)n1c1ccc(C)cc1
65) O=C(O)C1CC=CCC1c1nnc(c2ccncc2)n1c1ccccc1
66) O=C(O)C1CC=CCC1c1nnc(c2ccccc2)n1c1ccccc1
67) O=C1C=2CCCCC=2C(=O)N1/N=C(\Nc1ccccc1)c1ccccc1
68) Cc1ccc(cc1)N\C(=N/N1C(=O)C=2CCCCC=2C1=O)c1ccccn1
69) O=C(O)C=1CCCCC=1C(=O)N\N=C(/Nc1ccc(C)cc1)c1ccccn1
70) O=C(O)C=1CCCCC=1C(=O)N\N=C(/Nc1ccccn1)c1ccccn1
71) O=C(O)C=1CCCCC=1C(=O)N\N=C(/Nc1ccccc1)c1ccccn1
72) O=C(O)C=1CCCCC=1C(=O)N\N=C(/Nc1ccccc1)c1ccncc1
73) O=C(O)C=1CCCCC=1C(=O)N\N=C(/Nc1ccc(cc1)[N+]([O-])=O)c1ccccn1
74) O=C(O)C=1CCCCC=1c1nnc(c2ccccn2)n1c1ccc(C)cc1
75) O=C(O)C=1CCCCC=1c1nnc(c2ccccn2)n1c1ncccc1
76) O=C(O)C=1CCCCC=1c1nnc(c2ccccn2)n1c1ccccc1
77) O=C(O)C=1CCCCC=1c1nnc(c2ccncc2)n1c1ccccc1
78) O=C(O)C=1CCCCC=1C(=O)N\N=C(/Nc1ccc(C)cc1)c1ccncc1
79) O=C(O)C1CC=CCC1C(=O)N\N=C(/Nc1ccc(C)cc1)c1ccncc1
80) O=C(O)C1CC=CCC1c1nnc(c2ccncc2)n1c1ccc(C)cc1
81) O=C(O)\C=C(\C)c1nnc(c2ccccn2)n1c1ccc(C)cc1
82) O=C(O)C(/C)=C\c1nnc(c2ccccn2)n1c1ccc(C)cc1
83) O=C(O)/C=C\C(=O)NNC(Nc1ccccc1)c1ccncc1
84) O=C(O)/C=C\C(=O)NNC(Nc1ccc(C)cc1)c1ccncc1
85) [O-][N+](=O)c1ccc(cc1)NC(NNC(=O)\C=C/C(=O)O)c1ccccn1

Table **S1**. *S. aureus.* LASSO models were selected according to criteria. Fit statistics with F-values from ANOVA (n=83).

	Model 1 by Adj R^2	Model 2 by AIC	Model 3 by AICC	Model 4 by BIC	Model 5 by C(p)	Model 6 by SBC	Model 7 by ASE Val
Root MSE	258.93040	258.93040	267.52914	267.52914	258.93040	267.52914	282.05447
Dependent Mean	446.85542	446.85542	446.85542	446.85542	446.85542	446.85542	456.18966
R-Square	0.5160	0.5160	0.4542	0.4542	0.5160	0.4542	0.4983
Adj R^2	**0.4410**	0.4410	0.4032	0.4032	0.4410	0.4032	0.3645
AIC	1018.42750	**1018.42750**	1020.39968	1020.39968	1018.42750	1020.39968	725.76435
AICC	1023.70286	1023.70286	**1022.86543**	1022.86543	1023.70286	1022.86543	735.53179
BIC	940.17865	940.17865	938.43657	**938.43657**	940.17865	938.43657	678.53916
C(p)	10.08935	10.08935	10.90945	10.90945	**10.08935**	10.90945	8.60082
SBC	962.45359	962.45359	954.75040	954.75040	962.45359	**954.75040**	692.55011
ASE (Train)	57352	57352	64673	64673	57352	64673	61723
ASE (Validate)							**78022**
#effects	12	12	8	8	12	8	13
df1	11	11	7	7	11	7	12
df2	71	71	75	75	71	75	45
F	6.88	6.88	8.92	8.92	6.88	8.92	3.72
p	<.0001	<.0001	<.0001	<.0001	<.0001	<.0001	0.0006

Table **S2**. *S. aureus.* LAR models were selected according to criteria. Fit statistics with F-values from ANOVA (n=83).

	Model 1 by Adj R^2	Model 2 by AIC	Model 3 by AICC	Model 4 by BIC	Model 5 by C(p)	Model 6 by SBC	Model 7 by ASE Val
Root MSE	263.74805	265.90607	265.90607	265.90607	265.90607	290.59173	278.75705
Dependent Mean	446.85542	446.85542	446.85542	446.85542	446.85542	446.85542	444.43636
R^2	0.4978	0.4680	0.4680	0.4680	0.4680	0.3131	0.4879
Adj R^2	**0.4200**	0.4104	0.4104	0.4104	0.4104	0.2959	0.3715
AIC	1021.48771	**1020.27539**	1020.27539	1020.27539	1020.27539	1029.48304	686.06457
AICC	1026.76307	1023.33095	**1023.33095**	1023.33095	1023.33095	1029.99586	693.49314
BIC	942.22642	938.93134	938.93134	**938.93134**	938.93134	945.51502	630.06461
C(p)	12.68422	10.94076	10.94076	10.94076	**10.94076**	21.04900	24.60771
SBC	965.51380	957.04496	957.04496	957.04496	957.04496	**951.73957**	651.14524
ASE (Train)	59506	63039	63039	63039	63039	81391	62164
ASE (Validate)							**81791**
#effects	12	9	9	9	9	3	11
df1	11	8	8	8	8	2	10
df2	71	74	74	74	74	80	44
F	6.40	8.14	8.14	8.14	8.14	18.23	4.19
p	<.0001	<.0001	<.0001	<.0001	<.0001	<.0001	0.0004

Table S3. *S. aureus*. Stepwise models were selected according to criteria. Fit statistics with F-values from ANOVA (n=83).

	Model 1 by Adj R^2	Model 2 by AIC	Model 3 by AICC	Model 4 by BIC	Model 5 by C(p)	Model 6 by SBC	Model 7 by PRESS	Model 8 by ASE Val
Root MSE	265.36196	265.36196	265.36196	265.36196	265.36196	267.40487	265.36196	271.43058
Dependent Mean	446.85542	446.85542	446.85542	446.85542	446.85542	446.85542	446.85542	456.18966
R^2	0.4415	0.4415	0.4415	0.4415	0.4415	0.4256	0.4415	0.4321
Adj R^2	**0.4129**	0.4129	0.4129	0.4129	0.4129	0.4038	0.4129	0.4115
AIC	1016.30479	**1016.30479**	1016.30479	1016.30479	1016.30479	1016.63520	1016.30479	712.94957
AICC	1017.41006	1017.41006	**1017.41006**	1017.41006	1017.41006	1017.41442	1017.41006	713.70429
BIC	933.71305	933.71305	933.71305	**933.71305**	933.71305	933.73361	933.71305	656.38475
C(p)	6.71841	6.71841	6.71841	6.71841	**6.71841**	6.98840	6.71841	-6.04460
SBC	943.39900	943.39900	943.39900	943.39900	943.39900	**941.31057**	943.39900	659.13090
PRESS	6270484	6270484	6270484	6270484	6270484	6295028	**6270484**	4571450
ASE (Train)	66175	66175	66175	66175	66175	68059	66175	69864
ASE (Validate)								**81736**
#effects	5	5	5	5	5	4	5	3
df1	4	4	4	4	4	3	4	2
df2	78	78	78	78	78	79	78	55
F	15.41	15.41	15.41	15.41	15.41	9.51	15.41	20.93
p	<.0001	<.0001	<.0001	<.0001	<.0001	<.0001	<.0001	<.0001

Table S4. *N. corralina*. LASSO models were selected according to criteria. Fit statistics with F-values from ANOVA (n=50).

	Model 1 by Adj R^2	Model 2 by AIC	Model 3 by AICC	Model 4 by BIC	Model 5 by C(p)	Model 6 by SBC	Model 7 by ASE Val
Root MSE	195.25517	196.18997	196.18997	196.18997	195.25517	226.77668	177.39697
Dependent Mean	325.18000	325.18000	325.18000	325.18000	325.18000	325.18000	310.05556
R^2	0.3646	0.3432	0.3432	0.3432	0.3646	0.0000	0.4884
Adj R^2	**0.2587**	0.2516	0.2516	0.2516	0.2587	0.0000	0.3369
AIC	586.71306	**586.36720**	586.36720	586.36720	586.71306	595.38644	418.48752
AICC	591.21306	589.87940	**589.87940**	589.87940	591.21306	595.64176	427.28752
BIC	532.14881	531.99742	531.99742	**531.99742**	532.14881	543.72406	384.65953
C(p)	35.03786	35.36004	35.36004	35.36004	**35.03786**	60.64902	14.07613
SBC	550.00924	547.75136	547.75136	547.75136	550.00924	**545.29846**	394.73919
ASE (Train)	32025	33102	33102	33102	32025	50399	23602
ASE (Validate)							**36053**
#effects	8	7	7	7	8	1	9
df1	7	6	6	6	7	0	8
df2	42	43	43	43	42	49	27
F	3.44	3.74	3.74	3.74	3.44	.	3.22
P	0.0053	0.0044	0.0044	0.0044	0.0053	.	0.0106

Table S5. *N. corralina.* LAR models were selected according to criteria. Fit statistics with F-values from ANOVA (n=50).

	Model 1 by Adj R^2	Model 2 by AIC	Model 3 by AICC	Model 4 by BIC	Model 5 by C(p)	Model 6 by SBC	Model 7 by ASE Val
Root MSE	194.20908	194.20908	194.20908	194.20908	194.20908	206.62785	201.83112
Dependent Mean	325.18000	325.18000	325.18000	325.18000	325.18000	325.18000	340.84375
R^2	0.3564	0.3564	0.3564	0.3564	0.3564	0.2037	0.3525
Adj R^2	**0.2666**	0.2666	0.2666	0.2666	0.2666	0.1698	0.0876
AIC	585.35239	**585.35239**	585.35239	585.35239	585.35239	587.99816	381.68541
AICC	588.86458	588.86458	**588.86458**	588.86458	588.86458	588.88705	394.88541
BIC	531.20052	531.20052	531.20052	**531.20052**	531.20052	535.10932	350.70096
C(p)	33.92629	33.92629	33.92629	33.92629	**33.92629**	42.51841	18.44609
SBC	546.73655	546.73655	546.73655	546.73655	546.73655	**541.73423**	362.34277
ASE (Train)	32437	32437	32437	32437	32437	40133	28006
ASE (Validate)							**50818**
#effects	7	7	7	7	7	3	10
df1	6	6	6	6	6	2	9
df2	43	43	43	43	43	47	22
F	3.97	3.97	3.97	3.97	3.97	6.01	1.33
P	0.0030	0.0030	0.0030	0.0030	0.0030	0.0047	0.2775

Table **S6**. *N. corralina.* Stepwise models were selected according to criteria. Fit statistics with F-values from ANOVA (n=50).

	Model 1 by Adj R^2	Model 2 by AIC	Model 3 by AICC	Model 4 by BIC	Model 5 by C(p)	Model 6 by SBC	Model 7 by PRESS	Model 8 by ASE Val
Root MSE	149.15246	149.15246	149.15246	154.28996	149.15246	149.15246	149.15246	185.73874
Dependent Mean	325.18000	325.18000	325.18000	325.18000	325.18000	325.18000	325.18000	310.05556
R^2	0.6645	0.6645	0.6645	0.6316	0.6645	0.6645	0.6645	0.3146
Adj R^2	**0.5674**	0.5674	0.5674	0.5371	0.5674	0.5674	0.5674	0.2730
AIC	562.77506	**562.77506**	562.77506	565.46030	562.77506	562.77506	562.77506	417.02015
AICC	572.88617	572.88617	**572.88617**	573.89274	572.88617	572.88617	572.88617	418.31047
BIC	521.42191	521.42191	521.42191	**520.81639**	521.42191	521.42191	521.42191	379.99327
C(p)	10.44831	10.44831	10.44831	12.02883	**10.44831**	10.44831	10.44831	12.97785
SBC	533.71933	533.71933	533.71933	534.49256	533.71933	**533.71933**	533.71933	383.77070
PRESS	1533791	1533791	1533791	1602107	1533791	1533791	**1533791**	1405659
ASE (Train)	16907	16907	16907	18568	16907	16907	16907	31624
ASE (Validate)								**52769**
#effects	12	12	12	11	12	12	12	3
df1	11	11	11	10	11	11	11	2
df2	38	38	38	39	38	38	38	33
F	6.84	6.84	6.84	6.69	6.84	6.84	6.84	7.57
p	<.0001	<.0001	<.0001	<.0001	<.0001	<.0001	<.0001	0.0020

Table S7. *M. luteus.* LASSO models were selected according to criteria. Fit statistics with F-values from ANOVA (n=56).

	Model 1 by Adj R^2	Model 2 by AIC	Model 3 by AICC	Model 4 by BIC	Model 5 by C(p)	Model 6 by SBC	Model 7 by ASE Val
Root MSE	233.55646	235.18652	235.18652	235.18652	233.55646	235.18652	186.51987
Dependent Mean	324.76786	324.76786	324.76786	324.76786	324.76786	324.76786	315.41463
R^2	0.2658	0.2257	0.2257	0.2257	0.2658	0.2257	0.3949
Adj R^2	**0.1924**	0.1811	0.1811	0.1811	0.1924	0.1811	0.2192
AIC	674.43706	**673.41239**	673.41239	673.41239	674.43706	673.41239	480.27712
AICC	676.77040	674.61239	**674.61239**	674.61239	676.77040	674.61239	489.38056
BIC	614.56773	614.43655	614.43655	**614.43655**	614.56773	614.43655	436.06227
C(p)	36.78014	37.18813	37.18813	37.18813	**36.78014**	37.18813	27.70738
SBC	628.58917	623.51380	623.51380	623.51380	628.58917	**623.51380**	454.41284
ASE (Train)	48704	51362	51362	51362	48704	51362	26304
ASE (Validate)							**120404**
#effects	6	4	4	4	6	4	10
df1	5	3	3	3	5	3	9
df2	50	52	52	52	50	52	31
F	3.62	5.05	5.05	5.05	3.62	5.05	2.25
P	0.0071	0.0038	0.0038	0.0038	0.0071	0.0038	0.0455

Table S8. *M. luteus.* LAR models were selected according to criteria. Fit statistics with F-values from ANOVA (n=56).

	Model 1 by Adj R^2	Model 2 by AIC	Model 3 by AICC	Model 4 by BIC	Model 5 by C(p)	Model 6 by SBC	Model 7 by ASE Val
Root MSE	217.45418	231.39791	231.39791	217.45418	217.45418	231.39791	213.85843
Dependent Mean	324.76786	324.76786	324.76786	324.76786	324.76786	324.76786	273.75000
R^2	0.4145	0.2217	0.2217	0.4145	0.4145	0.2217	0.4662
Adj R^2	**0.2999**	0.2072	0.2072	0.2999	0.2999	0.2072	0.2814
AIC	669.76690	**669.70695**	669.70695	669.76690	669.76690	669.70695	432.58742
AICC	675.76690	670.16849	**670.16849**	675.76690	675.76690	670.16849	443.58742
BIC	611.11767	612.08309	612.08309	**611.11767**	611.11767	612.08309	387.75820
C(p)	28.42349	33.63740	33.63740	28.42349	**28.42349**	33.63740	46.44838
SBC	632.02041	615.75766	615.75766	632.02041	632.02041	**615.75766**	410.42261
ASE (Train)	38842	51633	51633	38842	38842	51633	33031
ASE (Validate)							**68963**
#effects	10	2	2	10	10	2	10
df1	9	1	1	9	9	1	9
df2	46	54	54	46	46	54	26
F	3.62	15.38	15.38	3.62	3.62	15.38	3.52
P	0.0018	0.0003	0.0003	0.0018	0.0018	0.0003	0.0315

Table S9. *M. luteus.* Stepwise models were selected according to criteria. Fit statistics with F-values from ANOVA (n=56).

	Model 1 by Adj R^2	Model 2 by AIC	Model 3 by AICC	Model 4 by BIC	Model 5 by C(p)	Model 6 by SBC	Model 7 by PRESS	Model 8 by ASE Val
Root MSE	208.50396	208.50396	210.96974	208.50396	208.50396	226.24808	208.50396	179.17653
Dependent Mean	324.76786	324.76786	324.76786	324.76786	324.76786	324.76786	324.76786	315.41463
R^2	0.4149	0.4149	0.3890	0.4149	0.4149	0.2559	0.4149	0.3155
Adj R^2	**0.3564**	0.3564	0.3410	0.3564	0.3564	0.2421	0.3564	0.2795
AIC	661.72889	**661.72889**	662.15459	661.72889	661.72889	667.18621	661.72889	471.33104
AICC	664.06223	664.06223	**663.86888**	664.06223	664.06223	667.64774	664.06223	472.44215
BIC	604.13307	604.13307	604.42844	**604.13307**	604.13307	609.72264	604.13307	428.66496
C(p)	20.37978	20.37978	21.22974	20.37978	**20.37978**	29.86805	20.37978	20.09710
SBC	615.88100	615.88100	614.28135	615.88100	615.88100	**613.23691**	615.88100	433.47176
PRESS	2829990	2829990	2842419	2829990	2829990	3050217	**2829990**	1484692
ASE (Train)	38816	38816	40534	38816	38816	49360	38816	29755
ASE (Validate)								**119037**
#effects	6	6	5	6	6	2	6	3
df1	5	5	4	5	5	1	5	2
df2	50	50	51	50	50	54	50	38
F	7.09	7.09	8.12	7.09	7.09	18.57	7.09	8.76
p	<.0001	<.0001	<.0001	<.0001	<.0001	<.0001	<.0001	0.0007

Table S10. *E. faecalis.* LASSO models were selected according to criteria. Fit statistics with F-values from ANOVA (n=56).

	Model 1 by Adj R^2	Model 2 by AIC	Model 3 by AICC	Model 4 by BIC	Model 5 by C(p)	Model 6 by SBC	Model 7 by ASE Val
Root MSE	333.65516	347.09687	347.09687	336.26777	333.65516	347.09687	327.81391
Dependent Mean	487.62500	487.62500	487.62500	487.62500	487.62500	487.62500	447.58537
R^2	0.2272	0.0000	0.0000	0.1297	0.2272	0.0000	0.0184
Adj R^2	**0.0760**	0.0000	0.0000	0.0614	0.0760	0.0000	-.0068
AIC	717.71632	**714.14660**	714.14660	714.36821	717.71632	714.14660	519.93015
AICC	723.71632	714.37302	**714.37302**	716.08250	723.71632	714.37302	520.57880
BIC	655.46927	656.79781	656.79781	**654.26550**	655.46927	656.79781	476.31353
C(p)	44.36297	49.98401	49.98401	44.49886	**44.36297**	49.98401	48.29459
SBC	679.96984	658.17195	658.17195	666.49497	679.96984	**658.17195**	480.35730
ASE (Train)	91446	118325	118325	102980	91446	118325	102220
ASE (Validate)							**156836**
#effects	10	1	1	5	10	1	2
df1	9	0	0	4	9	0	1
df2	46	55	55	51	46	55	39
F	1.50	.	.	1.90	1.50	.	0.73
p	0.1757	.	.	0.1247	0.1757	.	0.3979

Table S11. *E. faecalis.* LAR models were selected according to criteria. Fit statistics with F-values from ANOVA (n=56).

	Model 1 by Adj R^2	Model 2 by AIC	Model 3 by AICC	Model 4 by BIC	Model 5 by C(p)	Model 6 by SBC	Model 7 by ASE Val
Root MSE	322.86408	323.42089	347.09687	323.42089	323.42089	347.09687	343.41683
Dependent Mean	487.62500	487.62500	487.62500	487.62500	487.62500	487.62500	485.27778
R^2	0.2763	0.2581	0.0000	0.2581	0.2581	0.0000	0.3632
Adj R^2	**0.1348**	0.1318	0.0000	0.1318	0.1318	0.0000	0.1428
AIC	714.03414	**713.43148**	714.14660	713.43148	713.43148	714.14660	466.68883
AICC	720.03414	718.32037	**714.37302**	718.32037	718.32037	714.37302	477.68883
BIC	652.78723	652.34661	656.79781	**652.34661**	652.34661	656.79781	436.43878
C(p)	39.24883	39.15009	49.98401	39.15009	**39.15009**	49.98401	11.77187
SBC	676.28766	673.65965	658.17195	673.65965	673.65965	**658.17195**	444.52402
ASE (Train)	85627	87790	118325	87790	87790	118325	85175
ASE (Validate)							**81073**
#effects	10	9	1	9	9	1	10
df1	9	8	0	8	8	0	9
df2	46	47	55	47	47	55	26
F	1.95	2.04	.	2.04	2.04	.	1.65
p	0.0677	0.0614	.	0.0614	0.0614	.	0.1535

Table S12. *E. faecalis.* Stepwise models were selected according to criteria. Fit statistics with F-values from ANOVA (n=56).

	Model 1 by Adj R^2	Model 2 by AIC	Model 3 by AICC	Model 4 by BIC	Model 5 by C(p)	Model 6 by SBC	Model 7 by PRESS	Model 8 by ASE Val
Root MSE	249.48723	249.48723	249.48723	249.48723	249.48723	275.65884	249.48723	285.34377
Dependent Mean	487.62500	487.62500	487.62500	487.62500	487.62500	487.62500	487.62500	447.58537
R^2	0.5679	0.5679	0.5679	0.5679	0.5679	0.4266	0.5679	0.2944
Adj R^2	**0.4834**	0.4834	0.4834	0.4834	0.4834	0.3693	0.4834	0.2372
AIC	685.15789	**685.15789**	685.15789	685.15789	685.15789	692.99996	685.15789	510.39414
AICC	691.15789	691.15789	**691.15789**	691.15789	691.15789	695.33330	691.15789	512.10843
BIC	633.96303	633.96303	633.96303	**633.96303**	633.96303	636.26333	633.96303	466.52439
C(p)	8.93212	8.93212	8.93212	8.93212	**8.93212**	15.62334	8.93212	28.31132
SBC	647.41141	647.41141	647.41141	647.41141	647.41141	**647.15207**	647.41141	474.24843
PRESS	4378724	4378724	4378724	4378724	4378724	5048922	**4378724**	3644366
ASE (Train)	51129	51129	51129	51129	51129	67846	51129	73478
ASE (Validate)								**162627**
#effects	10	10	10	10	10	6	10	4
df1	9	9	9	9	9	5	9	3
df2	46	46	46	46	46	50	46	37
F	6.72	6.72	6.72	6.72	6.72	7.44	6.72	5.15
p	<.0001	<.0001	<.0001	<.0001	<.0001	<.0001	<.0001	0.0045

Table S13. *M. smegmatis.* LASSO models were selected according to criteria. Fit statistics with F-values from ANOVA (n=85).

	Model 1 by Adj R^2	Model 2 by AIC	Model 3 by AICC	Model 4 by BIC	Model 5 by C(p)	Model 6 by SBC	Model 7 by ASE Val
Root MSE	256.72843	257.34438	257.34438	257.34438	257.34438	279.08036	271.91516
Dependent Mean	389.55294	389.55294	389.55294	389.55294	389.55294	389.55294	377.43333
R^2	0.2646	0.2307	0.2307	0.2307	0.2307	0.0000	0.1055
Adj R^2	**0.1538**	0.1497	0.1497	0.1497	0.1497	0.0000	0.0575
AIC	1041.22690	**1039.05756**	1039.05756	1039.05756	1039.05756	1045.34903	738.51924
AICC	1046.35366	1042.03053	**1042.03053**	1042.03053	1042.03053	1045.49537	739.63035
BIC	955.59006	952.79916	952.79916	**952.79916**	952.79916	959.61954	677.92277
C(p)	26.31173	24.33659	24.33659	24.33659	**24.33659**	35.72430	12.74016
SBC	983.53872	974.04142	974.04142	974.04142	974.04142	**960.79168**	684.89662
ASE (Train)	56605	59214	59214	59214	59214	76970	69009
ASE (Validate)							**76803**
#effects	12	9	9	9	9	1	4
df1	11	8	8	8	8	0	3
df2	73	76	76	76	76	84	56
F	2.39	2.85	2.85	2.85	2.85	.	2.20
p	0.0138	0.0080	0.0080	0.0080	0.0080	.	0.0981

Table S14. *M. smegmatis.* LAR models were selected according to criteria. Fit statistics with F-values from ANOVA (n=85).

	Model 1 by Adj R^2	Model 2 by AIC	Model 3 by AICC	Model 4 by BIC	Model 5 by C(p)	Model 6 by SBC	Model 7 by ASE Val
Root MSE	264.91501	275.27694	277.12018	275.27694	264.91501	279.08036	278.36051
Dependent Mean	389.55294	389.55294	389.55294	389.55294	389.55294	389.55294	374.82143
R^2	0.2277	0.0502	0.0257	0.0502	0.2277	0.0000	0.0045
Adj R^2	**0.0989**	0.0271	0.0140	0.0271	0.0989	0.0000	0.0000
AIC	1047.39080	**1044.96798**	1045.13281	1044.96798	1047.39080	1045.34903	690.40212
AICC	1053.39080	1045.46798	**1045.42911**	1045.46798	1053.39080	1045.49537	690.86366
BIC	960.48168	958.36964	958.93163	**958.36964**	960.48168	959.61954	632.00131
C(p)	32.69537	33.76006	34.66879	33.76006	**32.69537**	35.72430	56.39626
SBC	992.14527	965.29593	963.01811	965.29593	992.14527	**960.79168**	636.45283
ASE (Train)	59447	73103	74989	73103	59447	76970	74717
ASE (Validate)							**81279**
#effects	13	3	2	3	13	1	2
df1	12	2	1	2	12	0	1
df2	72	82	83	82	72	84	54
F	1.77	2.17	2.19	2.17	1.77	.	0.25
p	0.0701	0.1208	0.1425	0.1208	0.0701	.	0.6220

Table **S15**. *M. smegmatis.* Stepwise models were selected according to criteria. Fit statistics with F-values from ANOVA (n=85).

	Model 1 by Adj R^2	Model 2 by AIC	Model 3 by AICC	Model 4 by BIC	Model 5 by C(p)	Model 6 by SBC	Model 7 by PRESS	Model 8 by ASE Val
Root MSE	270.26626	270.26626	270.26626	270.26626	270.26626	270.26626	270.26626	269.47613
Dependent Mean	389.55294	389.55294	389.55294	389.55294	389.55294	389.55294	389.55294	377.43333
R^2	0.0733	0.0733	0.0733	0.0733	0.0733	0.0733	0.0733	0.0900
Adj R^2	**0.0622**	0.0622	0.0622	0.0622	0.0622	0.0622	0.0622	0.0744
AIC	1040.87539	**1040.87539**	1040.87539	1040.87539	1040.87539	1040.87539	1040.87539	735.54348
AICC	1041.17169	1041.17169	**1041.17169**	1041.17169	1041.17169	1041.17169	1041.17169	735.97205
BIC	954.86238	954.86238	954.86238	**954.86238**	954.86238	954.86238	954.86238	675.17206
C(p)	29.01795	29.01795	29.01795	29.01795	**29.01795**	29.01795	29.01795	9.85481
SBC	958.76069	958.76069	958.76069	958.76069	958.76069	**958.76069**	958.76069	677.73217
PRESS	6386438	6386438	6386438	6386438	6386438	6386438	**6386438**	4554349
ASE (Train)	71325	71325	71325	71325	71325	71325	71325	70197
ASE (Validate)								**74998**
#effects	2	2	2	2	2	2	2	2
df1	1	1	1	1	1	1	1	1
df2	83	83	83	83	83	83	83	58
F	6.57	6.57	6.57	6.57	6.57	6.57	6.57	5.74
p	0.0122	0.0122	0.0122	0.0122	0.0122	0.0122	0.0122	0.0198

Table **S16**. *N. corralina.* Parameter estimates for LASSO models were optimized by seven fit criteria.

Parameter	Estimate b_i				Standardized estimate β_i				Mean selection percentage from Bootstrap
	Model 1 and 5 **	Model 2, 3 and 4**	Model 6	Model 7*	Model 1 and 5 **	Model 2, 3 and 4**	Mode l 6	Model 7*	
Intercept	1565.2074 24	1428.752 033	325.1800 00	1131.4220 88	0	0	0	0	
MW	-0.201253				- 0.02596 5				15.75
PASS_antiinflam	- 449.49409 8	- 371.8249 39		- 219.37778 6	- 0.33677 3	- 0.27858 1		- 0.18086 0	13.28
Acceptors_H	- 146.01472 8	- 136.6818 98			- 0.89179 3	- 0.83479 2			20.89
meltingTemp*R2_substit uent_2-pyridyl	0.393353	0.335244			0.13704 5	0.11680 0			27.11
meltingTemp*R2_substit uent_4-nitrophenyl	1.843860	1.705703		1.155768	0.52564 0	0.48625 5		0.33835 3	38.74
meltingTemp*R2_substit uent_phenyl	-0.624613	- 0.598389		-0.579565	- 0.26704 8	- 0.25583 7		- 0.27185 6	45.99
meltingTe*meltingTem	-0.003335	- 0.003431		-0.002771	- 0.21209 5	- 0.21820 0		- 0.19519 2	39.06

PASS_antieczematic				-207.957983				-0.253762	29.69
TPSA				-6.475011				-0.696661	47.20
R1_substituent_2-pyridyl				2.461317				0.005649	5.33
meltingTemp*R2_substituent_4-methylphenyl				0.255416				0.071773	29.27
R^2	0.3646	0.3432	0.0000	0.4884	0.3646	0.3432	0.0000	0.4884	
Adj R^2	0.2587	0.2516	0.0000	0.3369	0.2587	0.2516	0.0000	0.3369	
#effects	8	7	1	9	7	6	0	8	

Model 1 by Adj R^2, Model 2 by AIC, Model 3 by AICC, Model 4 by BIC, Model 5 by C(p), Model 6 by SBC, Model 7 by ASE Val, ** $p < 0.01$, * $p < 0.05$.

Table S17. *N. corralina.* Parameter estimates for LAR models were optimized by seven fit criteria.

Parameter	Estimate b_i			Standardized estimate β_i			Mean selection percentage from Bootstrap
	Model 1, 2, 3, 4 and 5**	Model 6**	Model 7	Model 1, 2, 3, 4 and 5**	Model 6**	Model 7	
Intercept	490.791006	258.196901	652.592448	0	0	0	
RmoExper	52.032957	59.227043	17.740635	0.161460	0.183783	0.063229	78.08
PASS_antieczematic	-304.981483	-147.842937	-267.471425	-0.355874	-0.172514	-0.343606	80.87
TPSA	-1.588766		-1.818948	-0.166473		-0.204866	38.70
R1_substituent_phenyl	20.793511		-39.063691	0.035584		-0.077648	31.08
meltingTemp*R2_substituent_4-methylphenyl	0.402160			0.111548			30.22
meltingTe*meltingTem	-0.001211		-0.003084	-0.077049		-0.196564	28.36
PASS_antibact			-70.797392			-0.038473	10.23
meltingTemp*R2_substituent_2-pyridyl			-0.058833			-0.020235	9.95
meltingTemp*R1_substituent_2-pyridyl			-0.086901			-0.036219	8.17
meltingTemp*R1_substituent_phenyl			0.763482			0.271799	3.55
R^2	0.3564	0.2037	0.3525	0.3564	0.2037	0.3525	
Adj R^2	0.2666	0.1698	0.0876	0.2666	0.1698	0.0876	
#effects	7	3	10	6	2	9	

Model 1 by Adj R^2, Model 2 by AIC, Model 3 by AICC, Model 4 by BIC, Model 5 by C(p), Model 6 by SBC, Model 7 ASE Val, ** $p < 0.005$.

Table S18. *M. luteus.* Parameter estimates for LASSO models were optimized by seven fit criteria.

Parameter	Estimate b_i			Standardized estimate β_i			Mean selection percentage from Bootstrap
	Model 1 and 5**	Model 2, 3,4 and 6**	Model 7*	Model 1 and 5**	Model 2, 3,4 and 6**	Model 7*	
Intercept	227.082714	166.975667	1284.554674	0	0	0	
MW	-0.308003			-0.033818			7.87
RmoExper	141.095422	113.080473		0.383279	0.307178		43.77
Donors_H	-10.570628		-91.164436	-0.044362		-0.478591	26.76
meltingTemp*R2_substituent_2-pyridyl	-0.151388	-0.145607	-0.200686	-0.044220	-0.042532	-0.070388	35.30
meltingTe*meltingTem	-0.000805		-0.000491	-0.048151		-0.036244	24.03
TPSA		-0.410882	-2.560736		-0.036767	-0.273558	48.50
PASS_antibact			39.502309			0.020738	5.14
PASS_antiinflam			-233.401251			-0.190011	10.04
PASS_antieczematic			-235.000140			-0.292914	19.31
perc_C			-6.882388			-0.142357	4.71
meltingTemp*R2_substituent_4-methylphenyl			0.420393			0.152422	34.76
R^2	0.2658	0.2257	0.3949	0.2658	0.2257	0.3949	
Adj R^2	0.1924	0.1811	0.2192	0.1924	0.1811	0.2192	
#effects	6	4	10	5	3	10	

Model 1 by Adj R^2, Model 2 by AIC, Model 3 by AICC, Model 4 by BIC, Model 5 by C(p), Model 6 by SBC, Model 7 by PRESS, Model 8 by ASE Val, * $p < 0.05$, ** $p < 0.01$.

Table S19. *M. luteus.* Parameter estimates for LAR models were optimized by seven fit criteria.

Parameter	Estimate b_i			Standardized estimate β_i			Mean selection percentage from Bootstrap
	Model 1, 4 and 5**	Model 2, 3 and 6**	Model 7*	Model 1, 4 and 5**	Model 2, 3 and 6**	Model 7*	
Intercept	679.049013	112.717663	322.879777	0	0	0	
MW	-1.337928		-0.957184	-0.146902		-0.116891	29.77
RmoExper	179.101225	118.093581	192.224985	0.486520	0.320796	0.569914	88.90
PASS_antiinflam	-65.555614			-0.043515			17.54
PASS_antieczematic	-132.206055			-0.136558			54.89
PASSp_antitumor	-205.642764		-265.326336	-0.087989		-0.121408	32.81
PASS_antituberculosi	230.279990		146.218794	0.117494		0.076520	37.37
Donors_H	-22.861903		-0.035985	-0.095945		-0.014536	36.20
meltingTemp*R1_substituent_phenyl	-0.142320		-0.015971	-0.042786		-0.005623	19.16
meltingTemp	-0.734339		1.071051	-0.119317		0.177482	10.10
meltingTe*meltingTem			-0.006311			-0.398602	20.91
R^2	0.4145	0.2217	0.4662	0.4145	0.2217	0.4662	
Adj R^2	0.2999	0.2072	0.2814	0.2999	0.2072	0.2814	

#effects	10	2	10	9	1	9	

Model 1 by Adj R^2, Model 2 by AIC, Model 3 by AICC, Model 4 by BIC, Model 5 by C(p), Model 6 by SBC, Model 7 by PRESS, Model 8 by ASE Val, * $p < 0.05$, ** $p < 0.005$.

Table **S20**. *E. faecalis.* Parameter estimates for LASSO models were optimized by seven fit criteria.

Paprameters	Estimate b_i				Standardized estimate β_i				Mean selection percentage from Bootstrap
	Model 1	Model 2, 3, and 6	Model 4	Model 7	Model 1	Model 2, 3, and 6	Model 4	Model 7	
Intercept	144.857149	487.625000	323.111454	572.864635	0	0	0	0	
RmoExper	88.459161		34.584429		0.179922		0.070343	0.179922	13.34
miLOGP	-39.988731				-0.118815			-0.118815	16.26
PASS_anti*PASS_antib	2303.306977		1684.609446		0.207126		0.151489	0.207126	41.79
perc_N	3.714414				0.033157				7.79
meltingTemp*R2_substituent_2-pyridyl	-0.150936				-0.033011				32.80
meltingTemp*R2_substituent_4-methylphenyl	0.741535		0.539976		0.162220				29.40
meltingTemp*R2_substituent_phenyl	0.978424		0.826485		0.277974				38.30
meltingTemp*R1_substituent_4-pyridyl	-0.220836				-0.048152		0.118126		4.36
meltingTe*meltingTem	0.002152				0.096386		0.234808		39.97
Acceptors_H				-18.344464				-0.073105	30.01
R^2	0.2272	0.0000	0.1297	0.0184	0.2272	0.0000	0.1297	0.0184	
Adj R^2	0.0760	0.0000	0.0614	-.0068	0.0760	0.0000	0.0614	-.0068	
#effects	10	1	5	2	9	0	4	1	

Model 1 by Adj R^2, Model 2 by AIC, Model 3 by AICC, Model 4 by BIC, Model 5 by C(p), Model 6 by SBC, Model 7 by ASE Val.

Table **S21**. *E. faecalis.* Parameter estimates for LAR models were optimized by seven fit criteria.

Parameters	Estimate b_i				Standardized estimate β_i				Mean selection percentage from Bootstrap
	Model 1	Model 2, 4 and 5	Model 3 and 6	Model 7	Model 1	Model 2, 4 and 5	Model 3 and 6	Model 7	
Intercept	1169.480460	1123.391774	487.625000	1125.271690	0	0	0	0	

MW	-2.937677	-2.722742		-3.320960	-0.241512	-0.223842		-0.275836	68.29
RmoExper	99.945361	90.967652		118.776532	0.203285	0.185025		0.239515	43.34
PASS_anti*PASS_antib	1320.065260	1187.328910		4045.720303	0.118707	0.106771		0.335699	34.97
PASS_antieczematic	-52.994244	-36.812216			-0.040986	-0.028471			23.01
PASSp_antitumor	-85.842044	-98.045954		-40.842220	-0.027502	-0.031411		-0.012711	30.70
PASS_antituberculosi	246.787305	238.239171		285.700451	0.094281	0.091015		0.101691	36.50
meltingTemp*R2_substituent_2-pyridyl	-0.123516	-0.067971			-0.027014	-0.014866			17.34
meltingTemp*R1_substituent_2-pyridyl	0.059717				0.015634				15.43
meltingTe*meltingTem	0.002743	0.002614		0.005906	0.122856	0.117097		0.253706	49.61
R2_substituent_4-nitrophenyl				-34.548571	0			-0.035205	6.50
meltingTemp*R2_substituent_phenyl				-0.111360				-0.030596	20.10
meltingTemp*R1_substituent_4-pyridyl				-0.155663				-0.031415	5.20
R^2	0.2763	0.2581	0.0000	0.3632	0.2763	0.2581	0.0000	0.3632	
Adj R^2	0.1348	0.1318	0.0000	0.1428	0.1348	0.1318	0.0000	0.1428	
#effects	10	9	1	10	9	8	0	9	

Model 1 by Adj R^2, Model 2 by AIC, Model 3 by AICC, Model 4 by BIC, Model 5 by C(p), Model 6 by SBC, Model 7 by ASE Val.

Table **S22**. *M. smegmatis.* Parameter estimates for LASSO models were optimized by seven fit criteria.

Parameter	Estimate b_i				Standardized estimate β_i				Mean selection percentage from Bootstrap
	Model 1*	Model 2, 3, 4 and 5*	Model 6	Model 7	Model 1*	Model 2, 3, 4 and 5*	Model 6	Model 7	
Intercept	567.476483	957.205261	389.552941	773.123825	0	0	0	0	
MW	-3.688674	-2.901385		-0.667514	-0.392103	-0.308415		-0.068771	74.36
PASS_antibact	108.184635				0.038401				20.07
PASS_antieczematic	-196.109210	-137.358410			-0.187693	-0.131464			52.97
perc_C	14.811843	7.367446			0.232604	0.115698			33.57
Acceptors_H	29.901267			-22.803541	0.142025			-0.100656	40.59

R1_substituent_2-pyridyl	-26.063150				-0.045783				28.19
R2_substituent_4-nitrophenyl	29.159139	57.636476			0.035278	0.069732			27.47
meltingTemp*R2_substituent_2-pyridyl	-1.494195	-1.317898		-0.522966	-0.400366	-0.353128		-0.138951	64.79
meltingTemp*R2_substituent_phenyl	-0.879063	-0.694533			-0.319515	-0.252443			61.11
meltingTemp*R1_substituent_4-pyridyl	-0.546309	-0.384363			-0.163355	-0.114930			49.83
meltingTe*meltingTem	0.003409	0.002611			0.207202	0.158709			54.79
R^2	0.2646	0.2307	0.0000	0.1055	0.2646	0.2307	0.0000	0.1055	
Adj R^2	0.1538	0.1497	0.0000	0.0575	0.1538	0.1497	0.0000	0.0575	
#effects	12	9	1	4	11	8	0	3	

Model 1 by Adj R^2, Model 2 by AIC, Model 3 by AICC, Model 4 by BIC, Model 5 by C(p), Model 6 by SBC, Model 7 by ASE Val, * $p < 0.05$.

Table S23. *M. smegmatis.* Parameter estimates for Stepwise models were optimized by eight fit criteria.

Parameter	Estimate b_i		Standardized estimate β_i		Mean selection percentage from Bootstrap
	Model 1, 2, 3, 4, 5, 6 and 7*	Model 8*	Model 1, 2, 3, 4, 5, 6 and 7*	Model 8*	
Intercept	534.682604	538.926425	0	0	
PASS_antitumor	-684.232146	-770.053682	-0.270800	-0.300081	19.39
R^2	0.0733	0.0900	0.0733	0.0900	
Adj R^2	0.0622	0.0744	0.0622	0.0744	
#effects	2	2	1	1	

Model 1 by Adj R^2, Model 2 by AIC, Model 3 by AICC, Model 4 by BIC, Model 5 by C(p), Model 6 by SBC, Model 7 by PRESS, Model 8 by ASE Val, * $p < 0.05$.

Table **S24**. *M. smegmatis.* Parameter estimates for LAR models were optimized by seven fit criteria.

Parameter	Estimate b_i					Standardized estimate β_i					Mean selection percentage from Bootstrap
	Model 1 and 5	Model 2 and 4	Model 3	Model 6	Model 7	Model 1 and 5	Model 2 and 4	Model 3	Model 6	Model 7	
Intercept	941.070974	438.615469	417.761089	389.552941	384.709700	0	0	0	0	0	
MW	-1.207300					-0.128335					65.04

PASS_anti*PASS_antib	440.744394					0.044047					49.71
PASS_antieczematic	-51.344029					-0.049141					58.83
PASSp_antitumor	-336.300973	-211.485435	-132.990879			-0.133098	-0.083700	-0.052634			73.69
PASS_antituberculosi	154.392357					0.079631					60.67
Donors_H	7.488815					0.027895					33.89
TPSA	-1.419848				-0.108924	-0.115155				-0.007328	23.69
R2_substituent_4-methylphenyl	80.805924					0.125622					53.43
meltingTemp*R2_substituent_2-pyridyl	-0.093006	-0.115940				-0.024921	-0.031066				53.60
meltingTemp*R2_substituent_4-nitrophenyl	0.688231					0.144440					46.43
meltingTemp*R1_substituent_4-pyridyl	-0.170319					-0.050928					48.11
meltingTemp*R1_substituent_phenyl	0.249893					0.066349					48.21
R^2	0.2277	0.0502	0.0257	0.0000	0.0045	0.2277	0.0502	0.0257	0.0000	0.0045	
Adj R^2	0.0989	0.0271	0.0140	0.0000	0.0000	0.0989	0.0271	0.0140	0.0000	0.0000	
#effects	13	3	2	1	2	12	2	1	0	1	

Model 1 by Adj R^2, Model 2 by AIC, Model 3 by AICC, Model 4 by BIC, Model 5 by C(p), Model 6 by SBC, Model 7 by ASE Val.

Table S25. Presence of main effects with positive and negative signs Tables 1-6 and S16-S24 for all considered bacterial strains *S. aureus* (SA), *M. luteus* (ML), *N. corallina* (NC), *E. faecalis* (EF), and *M. smegmatis* (MS).

variable	positive sign	negative sign	sum in row
MW	-	EF (2), SA (3), ML (3), MS (2), NC (2)	12
RMoExper	NC (2), EF (3), ML (3)	-	8
miLOGP	SA (1)	EF (1)	2
PASS_antibac	ML (1), MS (1), SA (1)	EF (1)	4
PASS_antibac*PASS_antibac	MS (1), EF (3)	-	4
PASS_antitubercul	EF (2), SA (1), ML (2), SA (1), MS (1)	-	7
PASS_antiinflam	SA (3)	EF (3), NC (2), ML (3)	11
PASS_antitumor	-	MS (2), SA (3), NC (1), ML (1), EF (1)	8
PASS_antieczematic	SA (3)	MS (2), NC (3), ML (3), EF (1)	12
TPSA	SA (1)	NC (2), MS (1), ML (1)	5
Acceptors_H	MS (1)	NC (1), EF (1)	3
Donors_H	MS (1)	ML (3), SA (1)	5
Perc_C	MS (1)	SA (3), ML (1)	5
Perc_N	SA (2), EF (1)	-	3
meltingTem*meltingTemp	EF (3), MS (1), SA (1)	NC (3), ML (2), SA (1)	11

meltingTemp	SA (1)	ML (1)	2
R1_substituent_2-pyridyl	MS (1)	NC (2)	3
R1_substituent_4-pyridyl	SA (1)	-	1
R1_substituent_phenyl	NC (1)	NC (1)	2
R2_substituent_nitrophenyl	MS (1)	SA (1)	2
R2_substituent_methylphenyl	MS (1), SA (1)	-	2

Table S26. Presence of main effects with positive and negative signs in Tables 1-6 and S16-S21 for four bacterial strains *S. aureus* (SA), *M. luteus* (ML), *N. corallina* (NC), and *E. faecalis* (EF).

variable	positive sign	negative sign	sum in row
MW	-	EF (2), SA (3), ML (3), NC (2)	10
RMoExper	NC (2), EF (3), ML (3)	-	8
miLOGP	SA (1)	EF (1)	2
PASS_antibac	ML (1), SA (1)	EF (1)	3
PASS_antibac*PASS_antibac	EF (3)	-	3
PASS_antitubercul	EF (2), SA (1), ML (2), SA (1)	-	6
PASS_antiinflam	SA (3)	EF (3), NC (2), ML (3)	11
PASS_antitumor	-	SA (3), NC (1), ML (1), EF (1)	7
PASS_antieczematic	SA (3)	NC (3), ML (3), EF (1)	10
TPSA	SA (1)	NC (2), ML (1)	4
Acceptors_H	-	NC (1), EF (1)	2
Donors_H	-	ML (3), SA (1)	3
Perc_C	-	SA (3), ML (1)	5
Perc_N	EF (1)	-	2
meltingTemp*meltingTemp	EF (3), SA (1)	NC (3), ML (2), SA (1)	10
meltingTemp	SA (1)	ML (1)	2
R1_substituent_2-pyridyl	-	NC (2)	2
R1_substituent_4-pyridyl	SA (1)	-	1
R1_substituent_phenyl	NC (1)	NC (1)	2
R2_substituent_nitrophenyl	-	SA (1)	1
R2_substituent_methylphenyl	SA (1)	-	1
